# Functional biology of the Steel syndrome founder allele and evidence for clan genomics derivation of *COL27A1* pathogenic alleles worldwide

**DOI:** 10.1038/s41431-020-0632-x

**Published:** 2020-05-06

**Authors:** Claudia Gonzaga-Jauregui, Gozde Yesil, Harikiran Nistala, Alper Gezdirici, Yavuz Bayram, Kalyan C. Nannuru, Davut Pehlivan, Bo Yuan, Johanna Jimenez, Yavuz Sahin, Ingrid S. Paine, Zeynep Coban Akdemir, Saathyaki Rajamani, Jeffrey Staples, John Dronzek, Kristen Howell, Jawid M. Fatih, Silvia Smaldone, Alan E. Schlesinger, Norman Ramírez, Alberto S. Cornier, Melissa A. Kelly, Robert Haber, Shek Man Chim, Kristy Nieman, Nan Wu, Johnathon Walls, Baylor-Hopkins Center for Mendelian Genetics, Regeneron Genetics Center, William Poueymirou, Chia-Jen Siao, V. Reid Sutton, Marc S. Williams, Jennifer E. Posey, Richard A. Gibbs, Simon Carlo, David H. Tegay, Aris N. Economides, James R. Lupski

**Affiliations:** 1 Regeneron Genetics Center, Regeneron Pharmaceuticals Inc., Tarrytown, NY 10591 USA; 2 Istanbul Faculty of Medicine Department of Medical Genetics, Istanbul University, 34093 Istanbul, Turkey; 3 Department of Medical Genetics, Kanuni Sultan Suleyman Training and Research Hospital, 34303 Istanbul, Turkey; 4 Department of Molecular and Human Genetics, Baylor College of Medicine, Houston, TX 77030 USA; 5 Department of Genetics and Genomic Sciences, Icahn School of Medicine at Mount Sinai, New York, NY 10029 USA; 6 Regeneron Pharmaceuticals Inc., Tarrytown, NY 10591 USA; 7 Department of Pediatrics, Division of Pediatric Neurology, Baylor College of Medicine, Houston, TX 77030 USA; 8 Medical Genetics, Genoks Genetics Center, 06570 Ankara, Turkey; 9 Texas Children’s Hospital, Houston, TX 77030 USA; 10 Department of Radiology, Baylor College of Medicine, Houston, TX 77030 USA; 11 Mayagüez Medical Center, Mayagüez, PR 00681 USA; 12 Genetics Section, San Jorge Children’s Hospital, San Juan, PR 00912 USA; 13 Ponce Health Sciences University, Ponce, PR 00716 USA; 14 Department of Pediatrics, Universidad Central del Caribe School of Medicine, Bayamon, PR 00960 USA; 15 Geisinger, Danville, PA 17822 USA; 16 Department of Orthopedic Surgery, Beijing Key Laboratory for Genetic Research of Skeletal Deformity, and Medical Research Center of Orthopedics, Peking Union Medical College Hospital, Peking Union Medical College & Chinese Academy of Medical Sciences, 100730 Beijing, China; 17 Human Genome Sequencing Center, Baylor College of Medicine, Houston, TX 77030 USA; 18 Department of Pediatrics, Division of Medical Genetics, Cohen Children’s Medical Center of Northwell Health, New Hyde Park, NY 11040 USA

**Keywords:** Disease genetics, Disease model

## Abstract

Previously we reported the identification of a homozygous *COL27A1* (c.2089G>C; p.Gly697Arg) missense variant and proposed it as a founder allele in Puerto Rico segregating with Steel syndrome (STLS, MIM #615155); a rare osteochondrodysplasia characterized by short stature, congenital bilateral hip dysplasia, carpal coalitions, and scoliosis. We now report segregation of this variant in five probands from the initial clinical report defining the syndrome and an additional family of Puerto Rican descent with multiple affected adult individuals. We modeled the orthologous variant in murine *Col27a1* and found it recapitulates some of the major Steel syndrome associated skeletal features including reduced body length, scoliosis, and a more rounded skull shape. Characterization of the in vivo murine model shows abnormal collagen deposition in the extracellular matrix and disorganization of the proliferative zone of the growth plate. We report additional *COL27A1* pathogenic variant alleles identified in unrelated consanguineous Turkish kindreds suggesting Clan Genomics and identity-by-descent homozygosity contributing to disease in this population. The hypothesis that carrier states for this autosomal recessive osteochondrodysplasia may contribute to common complex traits is further explored in a large clinical population cohort. Our findings augment our understanding of *COL27A1* biology and its role in skeletal development; and expand the functional allelic architecture in this gene underlying both rare and common disease phenotypes.

## Introduction

Steel et al. clinically described 23 patients with a novel orthopedic syndrome characterized by congenital bilateral hip and radial head dislocation, short stature, carpal coalitions, scoliosis, foot abnormalities, and mildly dysmorphic features [1, 2] [Steel Syndrome, STLS, MIM #615155]. Steel had initially observed this phenotypic presentation in seven patients from Puerto Rico in 1966 and reported his observations in 1973 as “The Puerto Rican syndrome” at an orthopedic surgery conference [1, 3]. Clinical and genetic evaluation of patients with STLS pointed to a distinct genetic syndrome different from other well-characterized skeletal dysplasias and connective tissue disorders. Targeted approaches evaluating genes associated with achondroplasia, Larsen syndrome, and Ehlers–Danlos syndrome were undertaken but unsuccessful at identifying a common molecular etiology for the characteristic features of STLS [2].

In 2015, we reported a single family of Puerto Rican origin with two affected siblings with a clinical diagnosis of STLS presenting with bilateral congenital hip dysplasia, scoliosis, short stature, under-ossification of femoral heads, bilateral capitate and hamate bone coalitions, bilateral fifth finger clinodactyly, midface hypoplasia, and pes planus. Exome sequencing (ES) of the two affected siblings identified a shared homozygous rare missense variant (c.2089G>C; p.Gly697Arg) in *COL27A1*, resulting in an amino acid substitution in the collagen type XXVII alpha 1 protein [4]. This variant had been reported before in population databases, but never in the homozygous state. Moreover, it did not appear to have any disease associated phenotypic consequences in young adult heterozygous carriers. We suggested this variant to be a founder allele in individuals of Puerto Rican descent segregating within a common haplotype [4].

Subsequent studies in large cohorts including multiethnic populations have identified the p.Gly697Arg variant in additional individuals, albeit at very low frequency. However, this allele is enriched, as hypothesized, primarily in individuals of Puerto Rican descent but also observed in other populations from the Caribbean, including the Dominican Republic and the island of St. Thomas, potentially due to migration within the region after the putative founder mutation event. It has been estimated that the carrier frequency for the *COL27A1* c.2089G>C (p.Gly697Arg) variant in Puerto Ricans is 1:51, that the mutation likely arose in a Native American haplotype background and that it drifted in the Puerto Rican population due to a strong population bottleneck about 9–14 generations ago [5], consistent with the demographic history of the Caribbean and the European colonization of the region [6].

Since our initial report, additional cases have been published linking novel rare recessive alleles in *COL27A1* with osteochondrodysplastic phenotypes, mainly in consanguineous pedigrees [7–11]. Patients presented with clinical findings overlapping those of the reported Puerto Rican patients with STLS, but also with additional features not previously recognized in STLS, namely congenital hearing loss and speech delay [7–11]. We now report on the biology of this disease through an in vivo mouse model of the most common disease-causing allele and the worldwide allelic architecture in clinical populations. Our data support a Clan Genomics hypothesis [12] for derivation of pathogenic variant alleles.

## Subjects and methods

### Samples

All patients included in this study were consented under an Institutional Review Board (IRB)-approved research protocol (H-29697) of Baylor College of Medicine. DiscovEHR participants are a subset of the Geisinger MyCode® Community Health Initiative. The MyCode® Community Health Initiative is a repository of blood, serum, and DNA samples from Geisinger patients that have been consented to participate in research and donate samples for broad research use, including genomic analyses that can be linked to de-identified electronic health record (EHR) information. DiscovEHR participants were consented in accordance with the Geisinger Institutional Review Board approved protocol, Study number 2006–0258.

### Targeted mutation testing of Steel syndrome c.2089G>C (p.Gly697Arg) variant

We obtained DNA from the affected probands, siblings, and unaffected parents and relatives for six of the originally reported Steel syndrome families [1, 2]. Targeted genotyping of the specific STLS variant [hg19 chr9:g.116958257G>C; COL27A1:c.2089G>C; p.Gly697Arg] was performed on all available individuals through sequence-specific PCR amplification and Sanger dideoxynucleotide sequencing. Genotyping was performed masked to affectation status and then matched to the available phenotype information. Similarly, we performed variant genotyping in an additional two patients of Puerto Rican ancestry that were referred to us for genomic studies through the Baylor-Hopkins Center for Mendelian Genomics (BHCMG) with a clinical presentation of STLS. A third family of Turkish ancestry where the proband (BAB10793) was given a clinical diagnosis of STLS was molecularly tested by Sanger sequencing of the entire *COL27A1* gene.

### Exome sequencing and analysis

We performed ES and analysis through the BHCMG initiative as previously described [13] in two probands with unspecified skeletal dysplasia from two separate consanguineous Turkish families. In addition, we performed ES in three affected adult siblings from a single family of Puerto Rican ancestry from New York (HOU2809) and with a clinical presentation of STLS in which the proband was shown to be positive for the STLS variant by targeted genotyping. We validated and segregated the candidate variants of interest in these three families through PCR amplification followed by Sanger sequencing. All identified novel *COL27A1* variants reported here have been deposited under accession ID SCV001190084 for public access through ClinVar (https://www.ncbi.nlm.nih.gov/clinvar/). In addition, genomic and phenotypic data for all subjects who have provided informed consent for data sharing in controlled-access databases have been submitted to the database of genotypes and phenotypes under accession phs000711.v5.

Sample preparation, ES, and sequence data production for the DiscovEHR participants were performed at the Regeneron Genetics Center as previously described [14].

BafCalculator was used to calculate a B**-**allele frequency at each variant position from ES data [15]. NMDEscPredictor was used to assess potential susceptibility to nonsense mediated decay (NMD) [16].

### Identity-by-decent (IBD) segment detection

We used KING 2.1.5 [17] with the –ibdseg command to identify all pairwise IBD segments between carriers of the hg19 chr9:g.116958257G>C variant using Illumina GSA genotyping array data for each sample. IBD segments on chromosome 9 were extracted from the KING results file and were visually tiled across the chromosome to illustrate the pileup of shared IBD that spanned the region containing the variant of interest. The shared IBD haplotype was defined as the minimum region of overlap across all variant carriers that have not been broken by recombination events.

### Animal care ethics statement and maintenance

All mice used in this study were housed under a specific pathogen free environment at the Regeneron Pharmaceuticals Inc. animal research facility. Autoclaved water and sterile mouse chow were provided *ad libitum*. All experimental protocols, anesthesia, imaging procedures, and tissue sampling procedures performed in this study were approved by the Regeneron Pharmaceuticals Inc. Institutional Animal Care and Use Committee.

### Generation of *Col27a1* mutant mice

To model the human p.Gly697Arg variant, a guanine-to-cytidine mutation, resulting in a p.Gly682Arg substitution (orthologous to the human variant), was introduced in the highly conserved triple-helical domain of the murine *Col27a1* gene. The mutation was introduced in exon 7 of 61 (exonID: ENSMUSE00001215121 [build GRCm38]) of the mouse gene using standard protocols for CRISPR/Cas9 gene editing and the VelociGene^©^ method [18, 19]. The final targeting vector was electroporated into C57BL/6N mouse embryonic stem cells, and selected via hygromycin resistance in the self-deleting cassette inserted in the downstream intron. Additional details on the targeting vector design and allele modification protocol can be found in Supplementary Methods. Mice carrying the introduced mutation were confirmed by Sanger dideoxynucleotide sequencing genotyping. Targeted, cassette-deleted knockin (KI) mice were bred to obtain desired genotypes. Homozygous and heterozygous KI mice cohorts derived from F1 breeding were generated for phenotypic evaluation.

### Mouse embryo phenotyping

X-ray µCT using iodine as a soft tissue enhancing contrast agent was performed as previously described [20]. Briefly, embryonic day 18.5 (E18.5) embryos were dissected and fixed using 4F1G fixative (4% PFA, 1% Glutaraldehyde in PBS) for 24 h. Embryos were skinned and then placed in a 1% iodine potassium iodide (IKI) solution for 24 h. Subsequently, embryos were embedded in agarose and imaged with an X-ray micro-computed tomography device (Zeiss VERSA XRM500). The data were acquired with an X-ray tube voltage of 100 kV, a current of 90 mA, an exposure time of 1300 ms, with 1025 views, and using the ×0.39 objective lens. Data were reconstructed at a pixel size of 20.6 µm using the XRadia software (XMReconstructor Cone Beam v10) and visualized using Amira 5.6.

### Growth and skeletal phenotyping

Mice were monitored for growth kinetics by recording weight at specified time points and gross skeletal phenotyping was performed by biweekly imaging with in vivo µCT performed as previously described [21]. Briefly, whole body µCT imaging was performed using a high speed in vivo μCT scanner (Quantum FX, PerkinElmer, Hopkinton, MA, USA). The X-ray source was set to a current of 88 μA, voltage of 90 kV. The CT imaging was visualized via 3D Viewer, existing software within the Quantum FX system. The field of view was 60, and voxel size was 240 μm. Mice were kept under anesthesia during scanning. Specifically, anesthesia was induced by keeping the mice under 2.5–3% Isoflurane with 1.5 l/min oxygen flow for 2–3 min and then positioned on the scan platform. Constant delivery of isoflurane was achieved via a nose cone connected to the scan platform. Following the scanning process, mice were recovered under a heating lamp and returned to their cages.

Following scanning, image processing steps were undertaken. Bone mineral density (BMD) and bone mineral content (BMC) were calculated from the µCT scanned image data using Analyze software package (AnalyzeDirect, Overland Park, KS, USA). BMC was calculated as BMD × Bone volume (mm^3^) × 1000. Volex gray intensities of the reconstructed images were transformed to linear Hounsfield units or linear attenuation coefficient, the scale is based on the data from images of the calibrating phantom, where air is −1000 HU and water 0 HU. Image segmentation was performed semiautomatically using the Volume Edit tools within the Analyze software package (AnalyzeDirect, Overland Park, KS, USA). Briefly, segmentation masks (object maps) were created using a combination of semiautomatic and manual techniques (object extraction, region growing, and thresholding tools). These segmentation results were then manually modified if necessary and quantified using the ROI tools.

### Histological analyses of mouse long bones

Post-natal day 1 (P1) long bone samples were fixed in 4% PFA at 4 °C for 16 h. Following fixation, samples were thoroughly washed with cold PBS multiple times and paraffin embedded without decalcification. Samples were sectioned at 3 µm until the plane of sectioning revealed the growth plate. Sections were then subjected to hematoxylin and eosin (H&E), Safranin-O or von Kossa staining according to standard protocols. Stained sections were imaged using Aperio Scanscope at ×40 magnification. For immunohistochemistry, all tibial samples underwent hyaluronidase digest (0.25% in Tris-buffered saline (TBS)) at 37 °C for 1 h. Samples were rinsed with TBS with 0.1% Triton-X. Subsequently, samples were incubated in blocking buffer, (10% goat serum, 3% bovine serum albumin (BSA) in TBS) for 1 h at room temperature. For Collagen II staining, sections were incubated overnight at 4 °C in a humidified chamber with primary antibody (II-II6B3, DSHB, 1:100 dilution). Sections were rinsed with TBS and incubated in goat anti mouse AF488 (A-11001, Invitrogen, 1:400 dilution) for 1 h at room temperature in 3% BSA in TBS. For Collagen X staining, sections were incubated overnight at 4 °C in a humidified chamber with primary antibody (X-AC9, DSHB, 1:100 dilution) diluted in 3% BSA in TBS. Sections were rinsed in TBS and incubated in goat anti mouse secondary AF594 (A-11005, Invitrogen, 1:400 dilution) for 1 h at room temperature in 3% BSA in TBS. Slides were rinsed and mounted with FluoroGel (17983–100, Electron Microscopy Sciences). Stained tissues were imaged using a Zeiss Axio Observer microscope.

## Results

### Confirmation of the *COL27A1* c.2089G>C (p.Gly697Arg) variant in additional Steel syndrome patients

**Fig. 1:**
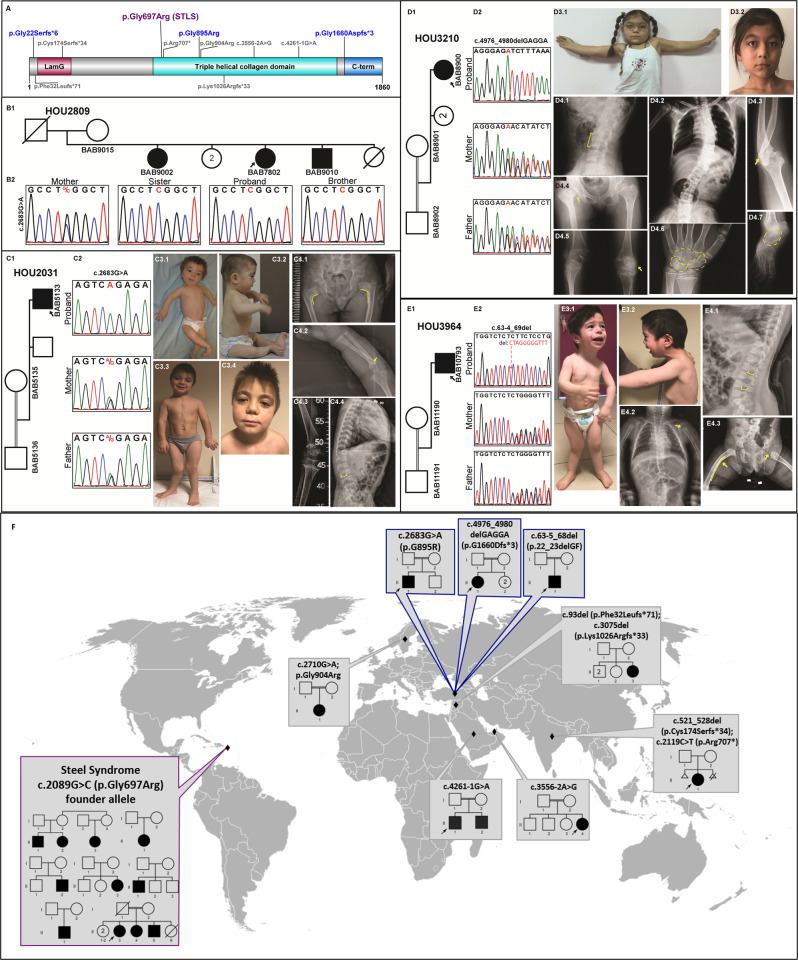
Spectrum of pathogenic allelic variation in *COL27A1* and novel variants identified in unrelated Turkish kindreds. **a** Domain structure of COL27A1 protein and variants identified to date associated with human disease phenotypes. Depicted are the Laminin G domain, the triple-helical domain characteristic of collagen proteins, and the fibrillar collagen C-term domain. The c.2089G>C (p.Gly697Arg) variant causative of Steel syndrome (STLS) occurs and likely disrupts the triple-helical domain, the c.2683G>A; p.(Gly895Arg) variant identified in Turkish patient (BAB5133) also occurs within the triple-helical domain potentially disrupting the structure of the protein. A novel frameshift variant c.4976_4980del; p.(Gly1660Aspfs*3) in a second Turkish patient (BAB8900) and an 11 bp deletion c.63-4_69del; p.(Gly22Serfs*6) in patient (BAB10793) are predicted to result in loss of function of COL27A1. **b** (B1 and B2) Pedigree and Sanger traces of Puerto Rican family referred for a clinical diagnosis of STLS. As expected affected individuals carry homozygous G>C change shown in red, resulting in the c.2089G>C (p.Gly697Arg) STLS variant. Unaffected mother is heterozygous for the variant. **c** (C1) Pedigree of family HOU2031. Index patient, BAB5133, is dark filled square and pointed with a black arrow. (C2) Segregation of identified missense variant [COL27A1:c.2683G>A; p.Gly895Arg] confirmed by Sanger sequencing in the family. Mutation is shown in red. Parents are heterozygous for the variant and patient is homozygous. (C3) Pictures of the index patient at different ages: C3.1 and C3.2 were taken at 15 months old showing midfacial hypoplasia, upturned nostril, short nasal bridge, and rhizomelic shortness. C3.3 and C3.4 pictures taken at 4 years old showing midfacial hypoplasia, upturned nostril, short nasal bridge, long philtrum, rhizomelic shortness, and genu valgum. (C4) X-rays of the patients showing: coxa valga (C4.1), radial head dislocation (C4.2), patellar dislocation (C4.3), and long vertebral pedicles (C4.4). **d** (D1) Pedigree of family HOU3210. Index patient, BAB8900, is indicated with a black arrow and dark filled circle. (D2) Segregation of identified frameshift variant [COL27A1:c.4976_4980del; p.(Gly1660Aspfs*3)] confirmed by Sanger sequencing in the family. The beginning of deletion is marked in red. As expected, individuals who carry the heterozygous deletion, BAB8901 (mother) and BAB8902 (father), have peak on peak appearance, while BAB8900 has normal appearing peaks due to homozygous deletion. (D3) Colored pictures of index case. D3.1 was taken at 9 years of age showing short nasal bridge, rhizomelic shortening of upper extremities, scoliotic posture. D3.2 was taken at age 11 showing the facial dysmorphic features including thick eyebrows, midface hypoplasia, and long philtrum. (D4) Skeletal survey of the index case showing multiple anomalies including tall vertebral body as pointed by yellow bracket (D4.1), severe scoliosis (D4.2), radial head dislocation (D4.3), hip dysplasia pointed by yellow arrow (D4.4), patellar dislocation (D4.5), and carpal (D4.6) and tarsal (D4.7) coalitions, merged bones are circled by dashed yellow lines. **e** (E1) Pedigree of family HOU3964, index BAB10793 is shown as black filled square. (E2) Segregation study of identified 11 bp deletion [COL27A1:c.63-4_69del; p.(Gly22Serfs*6)] in the family showing peak on peak signals in heterozygous parents and normal peaks for the homozygous deletion patient. (E3) Colored pictures of proband. E3.1 and E3.2 are pictures of the index patient showing typical dysmorphic features including midface hypoplasia, short nasal bridge, upturned nostril, long philtrum, rhizomelic shortness, and genu valga. (E4) Skeletal images of the index patient pointing long vertebral pedicles shown with yellow bracket (E4.1), glenoid hypoplasia pointed with yellow arrow (E4.2), femoral bowing shown with yellow line and hip dysplasia pointed with yellow arrow (E4.3). **f** Global distribution of pathogenic alleles identified to date in *COL27A1*. In purple the STLS c.2089G>C (p.Gly697Arg) founder allele identified in families of Puerto Rican ancestry. In blue, the three different pathogenic alleles identified in homozygosity in unrelated Turkish families reported here. In gray, previously reported disease associated variants in other populations [7–11]. Different disease-causing alleles homozygosed by consanguinity in the Turkish population and other populations around the world supports the Clan Genomics hypothesis.

To further investigate our founder allele hypothesis [4] that homozygosity for the c.2089G>C (p.Gly697Arg) variant in *COL27A1* is the molecular culprit for the clinically characterized Steel syndrome, we obtained samples from six of the originally reported and later on further clinically characterized patients with the defining syndrome STLS [1, 2] and their available family members. All of these families are from the island of Puerto Rico and reportedly non-consanguineous. We genotyped our previously reported STLS associated variant (hg19 chr9:g.116958257G>C; COL27A1:c.2089G>C; p.Gly697Arg) (Fig. 1a) in the *COL27A1* gene in all family members. Indeed, and in accordance with Mendelian expectations for an autosomal recessive (AR) disease trait, co-segregation of the c.2089G>C (p.Gly697Arg) variant with disease affectation status was observed in five of the six families genotyped (Supplementary Fig. 1). Retrospective review of the available clinical information for the proband of Family 4, that was negative for the *COL27A1* c.2089G>C (p.Gly697Arg) variant, showed that the patient only had bilateral hip dislocation and short stature but no apparent clinical manifestations involving her elbows, and no carpal coalitions, scoliosis, or foot deformities as in the other patients. In addition, her mother also had unilateral hip dislocation, suggesting a possible dominant disease trait or common complex trait of hip dislocation. ES of the proband did not reveal any additional variants of interest in *COL27A1* (data not shown) and the absence of the founder allele is inconsistent with a pseudodominant inheritance pattern that might arise from an affected individual marrying a carrier [22].

We also performed allele specific genotyping of the c.2089G>C (p.Gly697Arg) variant in suspected Steel syndrome families ascertained since our initial study, including a duo composed of the unaffected mother and her reportedly affected 7-year-old son with clinical and radiological findings of STLS, whom was recently published separately along with other two STLS patients [23]. The affected proband (BAB8472) was found to be homozygous for the STLS variant and his unaffected mother is, as anticipated, a heterozygous carrier. Separately, an affected 39-year-old female proband (BAB7802, Table 1) referred for a suspected clinical diagnosis of STLS, presented with short stature (1.47 m, −2.46 SD) and bilateral congenital hip dysplasia; joint instability with numerous dislocations involving the knees, elbows, and toes. There also was a history of motor delay; hearing loss that developed in the 4th decade of life; and difficulty with ambulation requiring a walker. She has severe scoliosis with lumbar lordosis, arthritis of the hip, knees, wrists, and spine, and describes progressive fatigue, joint pain, and disability manifesting during the last decade. The proband reported a loss of 7.62 cm in height over a 20 year period. Family history is positive for two similarly affected adult siblings with short stature, congenital hip and knee dislocations, scoliosis, and hearing loss (Supplementary Information). The family (HOU2809) is of Puerto Rican ancestry with no reported consanguinity and additionally composed of unaffected parents of normal stature (father 1.63 m [−1.93 SD] and mother 1.60 m [−0.51 SD]) and two other unaffected siblings of normal height. ES and targeted mutation testing in all affected siblings confirmed a molecular diagnosis of STLS due to homozygosity for the c.2089G>C (p.Gly697Arg) variant in *COL27A1* (Fig. 1b). Review of the ES data for rare variation in genes associated with hearing loss revealed one heterozygous shared variant in *CLCNKA* (NM_004070.3:c.820G>A; p.Asp274Asn). *CLCNKA* has been associated with Bartter syndrome type 4b [MIM #613090] through digenic inheritance in combination with *CLCNKB* variants. However, no additional shared single nucleotide or copy number variants in *CLCNKB* were identified, and there is no clinical history of salt wasting, a major clinical feature of Bartter syndrome (MIM #613090), in the affected siblings. Given the absence of digenic inheritance of *CLCNKA* and *CLCNKB* variants, and the incomplete phenotypic overlap in this family, the observed hearing loss in the affected siblings cannot be attributed to the identified *CLCNKA* variant alone.

**Table 1:** Clinical characteristics of STLS patients homozygous for the p.Gly697Arg variant in COL27A1.

	Steel et al. [1]	Flynn et al. [2]^a^	Gonzaga-Jauregui et al. [4]	Belbin et al. [5]	Amlie-Wolf et al. [23]	STLS patients^b^	HOU2809	GHS01
						(this study)	(this study)	(this study)
Ethnicity	Puerto Rican	Puerto Rican	Puerto Rican	Puerto Rican	Puerto Rican	Puerto Rican	Puerto Rican	Hispanic
COL27A1 Variant(s)	NA	NA	c.2089G>C; p.Gly697Arg	c.2089G>C; p.Gly697Arg	c.2089G>C; p.Gly697Arg	c.2089G>C; p.Gly697Arg	c.2089G>C; p.Gly697Arg	c.2089G>C; p.Gly697Arg
Consanguinity	No	No	No	No	No	No	No	No
Gender	8 M; 15 F	17 M; 15 F	1 M; 2 F	2 M; 3 F	2 M; 1 F	3 M; 2 F	1 M; 2 F	F
Zygosity	NA	NA	HMZ	HMZ	HMZ	HMZ	HMZ	HMZ
Age at evaluation	7 y (0.5–17 y)	17.8 y (3.7–50 y)	14 and 12 y	51.6 y (34–74 y)	2 months, 7 and 14 y	NA	33, 39 and 48 y	39
***Steel syndrome features***								
Congenital bilateral hip dislocation	23/23 (100%)	32/32 (100%)	3/3 (100%)	5/5 (100%)	3/3 (100%)	5/5 (100%)	3/3 (100%)	+
Short stature	20/23 (87%)	32/32 (100%)	3/3 (100%)	5/5 (100%)	2/3 (66%)	5/5 (100%)	3/3 (100%)	+
Carpal coalition	39/46 (85%)	47/64 (73%)	3/3 (100%)	2/5 (40%)	NA	5/5 (100%)	NA	NA
Radial head dislocation	33/46 (72%)	29/32 (91%)	1/3 (33%)	1/5 (20%)	2/3 (66%)	5/5 (100%)	3/3 (100%)	+
Scoliosis	15/23 (65%)	17/32 (53%)	3/3 (100%)	2/5 (40%)	3/3 (100%)	5/5 (100%)	3/3 (100%)	+
Foot deformity	Pes cavus (35%)	Pes cavus (34%)	−	−	Metatarsus adductus (1/3)	NA	Pes planus; Displacement of cuneiform	+
Vertebral anomalies	4/23 (17%)	3/32 (10%)	1/3 (33%)	3/5 (60%)	NA	NA	+	+
***Other skeletal and radiological findings***								
Digit anomalies	NA	Brachydactyly	Bilateral 5th finger clinodactyly	NA	NA	NA	Brachydactyly	NA
Lumbar lordosis	+	+	+	1/5 (20%)	NA	+	+	NA
Coxa vara	NA	NA	+	NA	NA	NA	NA	NA
Genu Valgum	NA	NA	−	NA	NA	NA	+	NA
Vertical talus	NA	1/32 (3%)	−	NA	NA	NA	−	NA
Acetabular dysplasia	NA	+	+	NA	1/3	NA	+bilateral	NA
Joint anomalies	No hyperlaxity	No hyperlaxity	Decreased elbow extension	NA	Decreased elbow extension; multiple joint pain	NA	Decreased elbow extension; patellar, elbow, knee, and toe dislocations; joint pain	Elbow deformity; Multiple joint pain
Other skeletal anomalies	Pectus carinatum/excavatum 3/23 (13%)	NA	Under ossified capital femoral epiphyses	NA	Pectus excavatum (2/3)	NA	−	NA
***Facial features***								
Long oval face	NA	+	+	NA	NA	NA	NA	NA
Prominent forehead	NA	+	+	NA	NA	NA	NA	NA
Frontal bossing	NA	+	+	NA	+	NA	NA	NA
Arched/thick eyebrows	NA	NA	−	NA	+	NA	NA	NA
Midface hypoplasia	NA	+	+	NA	+	NA	NA	NA
Hypertelorism	NA	+	−	NA	+	NA	+	NA
Short and broad nasal bridge	NA	+	+	NA	+	NA	+	NA
Anteverted nares	NA	NA	+	NA	NA	NA	NA	NA
Long philtrum	NA	NA	+	NA	NA	NA	NA	NA
High-arched palate	NA	+	−	NA	+	NA	NA	NA
Ears	NA	Small, posteriorly rotated ears	−	NA	NA	NA	NA	NA
Other dysmorphic features	NA	NA	−	NA	NA	NA	NA	NA
***Additional clinical features***								
Developmental Delay	NA	Normal developmental milestones (100%)	Normal development and intelligence	NA	Normal development and intelligence (2/3)	NA	Normal development and intelligence	NA
Hearing loss	NA	−	−	−	−	NA	3/3; Bilateral dx >30 y	Bilateral dx >30 y
Walking difficulties	NA	NA	−	NA	+	NA	+	+
Other clinical features	NA	NA	NA	Some individuals with arthritis, osteoporosis or osteopenia	Microcephaly and failure to thrive (1 pt); Osteopenia (1 pt)	NA	Arthritis and joint arthralgia, myopia	Failure of total hip arthroplasty

*M* male, *F* female, *y* year old, *HMZ* homozygous, *CMPD HTZ* compound heterozygous, *NA* not available/assessed.

^a^The report by Flynn et al. [2] includes 18 of the 23 patients originally reported by Steel et al. [1] plus additional 14 patients.

^b^This study reports the molecular findings of 5 of the original STS patients reported by Steel et al. [1] and reviewed by Flynn et al. [2].

### Molecular characterization of an allelic series at the *COL27A1* locus

Independently, we performed ES and family-based genomic analyses of two different Turkish families in which the affected probands had uncharacteristic skeletal dysplasias. The first family (HOU2031) is composed of an affected 4-year-old male proband (BAB5133) and his unaffected consanguineous parents (Table 2, Fig. 1c, and Supplementary Information). Parental consanguinity was elicited by historical report and further evidenced by a total genomic absence of heterozygosity (AOH) of 422.948 Mb in the proband calculated from ES data. The first clinical examination for dysmorphic features occurred at age 1 year and 3 months and revealed wooly sparse hair, prominent forehead, short upturned nose, long philtrum, torticollis, rhizomelia of the upper limbs, bowing of femurs, rocker bottom feet, pes planovalgus, and joint laxity. The proband was short for age, had mild developmental delay and unilateral conductive deafness presumed secondary to recurrent infections. X-rays showed bowing bent femurs, delayed epiphyseal ossification, and hypoplastic clavicles and prominent metaphyseal irregularity. Lumbar kyphosis was also noted. X-rays of lateral spine and forearms at 5 months of age revealed tall vertebral bodies, long vertebral pedicles, and possibly dislocated radial heads.

**Table 2:** Clinical characteristics of patients with biallelic protein altering variants in COL27A1.

	STLS patients	Gariballa et al. [7]	Kotabagi et al. [8]	Maddirevula et al. [9]	Thuresson et al. [10]	Pölsler et al. [11]	BAB5133	BAB8900	BAB10793
							(this study)	(this study)	(this study)
Ethnicity	Puerto Rican	Emirati	Indian	Saudi	Unspecified	Syrian	Turkish	Turkish	Turkish
COL27A1 Variant(s)	c.2089G>C; p.Gly697Arg	c.3556–2A>G	c.521_528del; p.Cys174Serfs*34|c.2119C>T; p.Arg707*	c.4261–1G>A	c.2710G>A; p.Gly904Arg	c.93del; p.Phe32Leufs*71|c.3075del; p.Lys1026Argfs*33	c.2683G>A; p.Gly895Arg	c.4976_4980 del; p.Gly1660Aspfs*3	c.63–4_69del; p.Gly22Serfs*6
Consanguinity	No	Yes	No	Yes	Yes	No	Yes	Yes	Yes
Gender	–	F	F	M	F	F	M	F	M
Zygosity	HMZ	HMZ	CMPD HTZ	HMZ	HMZ	CMPD HTZ	HMZ	HMZ	HMZ
Age at evaluation	–	3 y	5 y	5 y	10 y	9 y	6 y	11 y	5–21 months
***Steel syndrome features***									
Congenital bilateral hip dislocation	100%	+	+	+	+	+	+	+	+
Short stature	95%	NA	+	+	+	+	+	+	+
Carpal coalition	77%	NA	+	NA	NA	NA	−	+	−
Radial head dislocation	65%	+	+	NA	NA	+	(Suspected)	+	+
Scoliosis	85%	NA	+	+	+	+	+	+	−
Foot deformity	~50%	−	Pes equinovarus	+	Pes cavus		Pes equinovarus; Pes planovalgus	Pes equinovarus; Pes cavus	+ (Mild)
Vertebral anomalies	~65%	+	NA	NA	+	+	Torticollis	+	+
***Other skeletal and radiological findings***									
Digit anomalies	+	None noted	Bilateral 5th finger clinodactyly; Partial syndactyly of fingers and toes	NA	NA	NA	5th finger clinodactyly	5th finger clinodactyly	5th finger clinodactyly
Lumbar lordosis	+	NA	+	NA	NA	+	Kyphosis	+	+
Coxa vara	+	+	+	NA	+	+	NA	NA	+
Genu valgum	+	+; femoral bowing	+	NA	NA	NA	+; femoral bowing	+	+; femoral bowing
Vertical talus	NA	NA	Bilateral	NA	NA	+	NA	+	NA
Acetabular dysplasia	+	+	+	NA	NA	NA	NA	+	NA
Joint anomalies	+	NA	NA	NA	NA	+	Hyperlaxity	Limited elbow and hip movement; patellar dislocation	NA
Other skeletal anomalies	+	Rhizomelic shortening of upper extremities	Small and under ossified femoral epiphyses	NA	Rhizomelic shortening of upper extremities, femoral collum pseudoarthrosis, sacral dimple	Thoracolumbar kyphosis	Delayed epiphyseal ossification, hypoplastic clavicles, metaphyseal irregularity	Rhizomelic shortening of upper extremities	Rhizomelic shortening of upper extremities
***Facial features***									
Long oval face	+	NA	+	+	NA	NA	+	+	NA
Prominent forehead	+	NA	+	+	NA	+	+	−	+
Frontal bossing	+	NA	+	NA	NA	NA	−	NA	NA
Arched/thick eyebrows	+	+	NA	NA	NA	+	+	+	−
Midface hypoplasia	+	+	NA	NA	NA	+	+	+	+
Hypertelorism	+	NA	Telecanthus	NA	+	NA	NA	NA	NA
Short and broad nasal bridge	+	+	+	+	NA	+	+	+	+
Anteverted nares	+	+	+	NA	NA	NA	+	+	+
Long philtrum	+	+	+	NA	NA	+	+	+	+
High-arched palate	+	NA	+	NA	NA	NA	NA	NA	NA
Ears	NA	NA	Bilateral low set ears	Bilateral low set ears	NA	NA	NA	NA	NA
Other dysmorphic features	NA	NA	Retrognathia	Conical teeth	NA	NA	Wooly sparse hair	NA	NA
***Additional clinical features***									
Developmental Delay	Normal development and intelligence	Normal development but speech delay	Motor developmental and speech delay	NA	+	Motor developmental and speech delay	+	+	+
Hearing loss	Bilateral hearing loss of adult onset	Severe, bilateral, sensorineural	+	NA	+; bilateral	+; bilateral	Unilateral, conductive	+; bilateral	+
Walking difficulties	+	NA	+	+	NA	+	+	+	+
Other clinical features	NA	NA	NA	Inguinal hernia and undescended testicles	Mild intellectual disability. Homozygous FRMD4A VUS	Bilateral colobomata, hyperopia and strabismus	Ventricular septal defect, pulmonary hypertension	NA	NA

*M* male, *F* female, *y* year old, *HMZ* homozygous, *CMPD HTZ* compound heterozygous, *NA* not available/assessed.

ES and rare variant family based genomic analyses of the proband identified a homozygous variant in exon 19 of *COL27A1* [hg19 chr9:g.116999951G>A; COL27A1:c.2683G>A; p.(Gly895Arg)]. The variant is novel and absent from our internal databases, nor has it been reported in any available public database. The variant is predicted to be likely deleterious or damaging due to high conservation of the residue and predicted to be function altering according to Polyphen2 and Mutation Taster. Similar to the STLS c.2089G>C (p.Gly697Arg) variant, this variant likely affects the collagen triple-helical domain of the protein (Fig. 1a). On follow-up, a detailed skeletal examination at 6 years of age did not identify any carpal coalitions.

The second Turkish family (HOU3210) is composed of an affected female proband (BAB8900, Table 2 and Fig. 1d), unaffected consanguineous parents and two reportedly unaffected siblings. The proband had an uncomplicated prenatal and birth history. Physical examination at age 11 years revealed bilateral hearing loss, motor developmental delay, limited elbow movement, scoliosis, bilateral limited hip movement, genu valgum, patellar dislocation, and pes equinovarus. Skeletal survey revealed radial head dislocation, carpal, and tarsal coalitions, clinodactyly, tall vertebral bodies, bilateral hip dysplasia (right worse than left), and partial knee dislocation (right worse than left) and pes cavus. ES analysis identified a homozygous frameshift deletion in exon 58 [hg19 chr9:g.117068837_117068841del; COL27A1:c.4976_4980del; p.(Gly1660Aspfs*3)] that is predicted to result in the introduction of a premature stop codon with likely degradation of the altered *COL27A1* mRNA transcript through NMD [9] (Fig. 1a).

A third family (HOU3210), also from Turkey, includes a 21-month-old male proband (BAB10793, Table 2 and Fig. 1e) born to unaffected consanguineous parents. He was initially evaluated at age 5 months, and classical clinical features such as scoliosis, hip dislocation, and short stature prompted a suspected clinical diagnosis of STLS and subsequent molecular evaluation by Sanger sequencing of the entire *COL27A1* gene to look for potential protein altering variants. He was found to have a homozygous 11 bp deletion involving the intron1/exon2 boundary [hg19 chr9:g.116924991_116925001del; c.63-4_69del; p.(Gly22Serfs*6)] deleting the splice acceptor site of exon 2 and introducing an early stop codon in the mRNA transcript that is likely degraded through NMD. Skeletal survey at 18 months old showed glenoid fossa hypoplasia, radial head dislocation, tall vertebral bodies, long vertebral pedicles, left hip dislocation, and dysplasia as evidenced by an acetabular roof that is irregular and at an increased angle (coxa vara), bowing of the femurs (right worse than left). Radiographs of the feet were not available for review. This proband additionally has developmental delay and hearing loss.

**Fig. 2:**
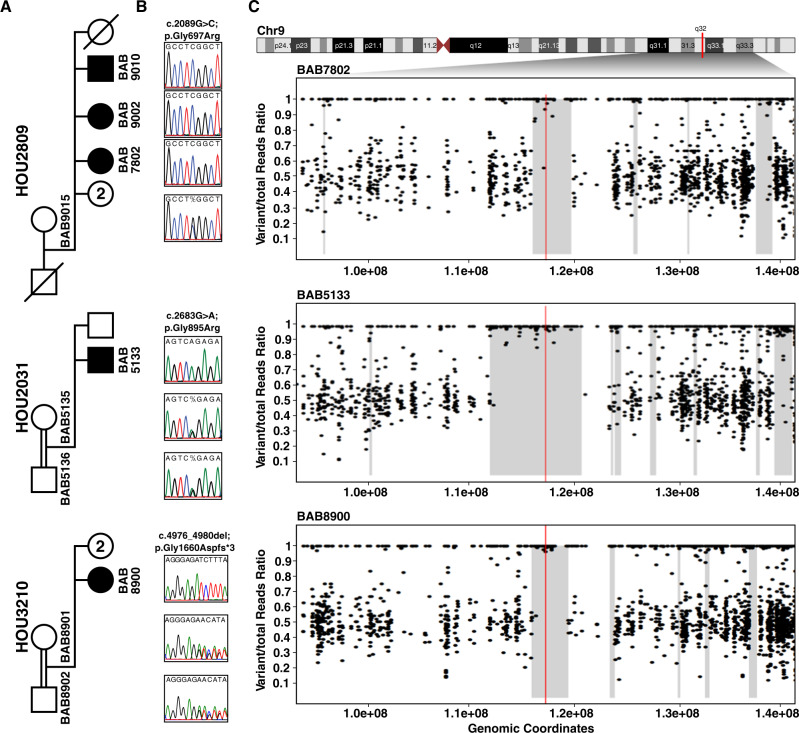
Runs of homozygosity in pedigrees segregating pathogenic variants in *COL27A1*. **a** The pedigree of the families with family ID (HOU #) above and individual ID # below in each individual. Affected individuals are shown as black-filled symbols. **b** Segregation study of the *COL27A1* variants in individuals from each of the different pedigrees. **c** (Top) Ideogram of chromosome 9. (Bottom) Zoomed-in views (dashed line) of B-allelic frequency plots encompassing the *COL27A1* region. The coordinates of *COL27A1* variants are indicated by vertical red lines embedded within an AOH block shown as gray regions.

Of note, the variants identified in *COL27A1* in these three Turkish individuals with skeletal dysplasia have not been observed in any publicly available variant databases nor in ~1100 exomes from Turkish individuals sequenced as part of the BHCMG initiative. Further, and likely due to consanguinity in the parents of the probands, these rare variants map within regions of AOH ranging from 3.68 to 8.83 Mb in length, as delineated from unphased ES variant data and presumably reflecting runs of homozygosity resulting from IBD in the personal genomes of these individuals (Fig. 2). The rarity of the variants identified and the total genome-wide AOH, calculated from WES data, for the Turkish probands is consistent with a clan genomics IBD and transmission genetics homozygosity of a recent mutation in the family or clan versus an older founder allele homozygosed [24] by geographic or population isolation [25, 26] (Fig. 1f).

### Phenotypic characterization of *COL27A1* c.2089G>C (p.Gly697Arg) variant carriers from a large clinical cohort and haplotype refinement

Through the Geisinger–Regeneron DiscovEHR collaboration that pairs genomic data with EHR information [14], we were able to identify 28 adult heterozygous carriers of the c.2089G>C (p.Gly697Arg) variant in *COL27A1* and 1 homozygous female. Twenty-three of these 29 individuals have self-reported ancestry information of being Hispanic, consistent with the known genetic background for the STLS variant, although without further detail available. Review of the EHR information for the homozygous individual revealed a clinical presentation consistent with STLS including history of congenital hip dysplasia, short stature (1.50 m), scoliosis, radial head subluxation, and foot abnormalities. She also has diagnoses of tinnitus and bilateral sensorineural hearing loss first recorded at 38 years of age. Furthermore, her EHR documents a history of surgical interventions and failures for hip arthroplasty accompanied by chronic pain affecting multiple joints (Table 1 and Supplementary Table 1), consistent with previous reports of untoward surgical outcomes in STLS patients [2].

The sizable number of heterozygous carrier individuals allowed us to further explore the possible contribution of the carrier status to common skeletal and joint phenotypes. While not statistically significant, manual exploration of the EHR data for these individuals revealed some recurrent clinical diagnoses of cervicalgia (ICD9 723.1), arthropathy (ICD9 716.9), osteoarthrosis (ICD9 715.00), and pain in back, limbs, and joints across the 28 heterozygous STLS variant carriers (Supplementary Table 1). In addition, whole-genome IBD segment mapping across carriers of the c.2089G>C (p.Gly697Arg) variant showed that all carriers share the region in which the variant is embedded IBD. Through this analysis we were also able to further refine the region containing this founder variant to a 2.682 Mb common haplotype (Supplementary Fig. 2).

### In vivo mouse modeling of the Steel syndrome c.2089G>C (p.Gly697Arg) variant

To garner functional evidence for pathogenicity, and better understand the pathophysiological effects of the identified c.2089G>C (p.Gly697Arg) variant and disease trait biology in individuals with STLS, we engineered a KI mouse model. The human *COL27A1* and mouse *Col27a1* orthologous genes are 84% identical at the nucleotide level and the encoded proteins are 90% identical and 94% similar at the amino acid level. To model the human c.2089G>C (p.Gly697Arg) variant, the equivalent G>C mutation was introduced in the murine *Col27a1* gene resulting in a p.Gly682Arg substitution (orthologous to the human variant) in the mouse (Supplementary Fig. 3).

**Fig. 3:**
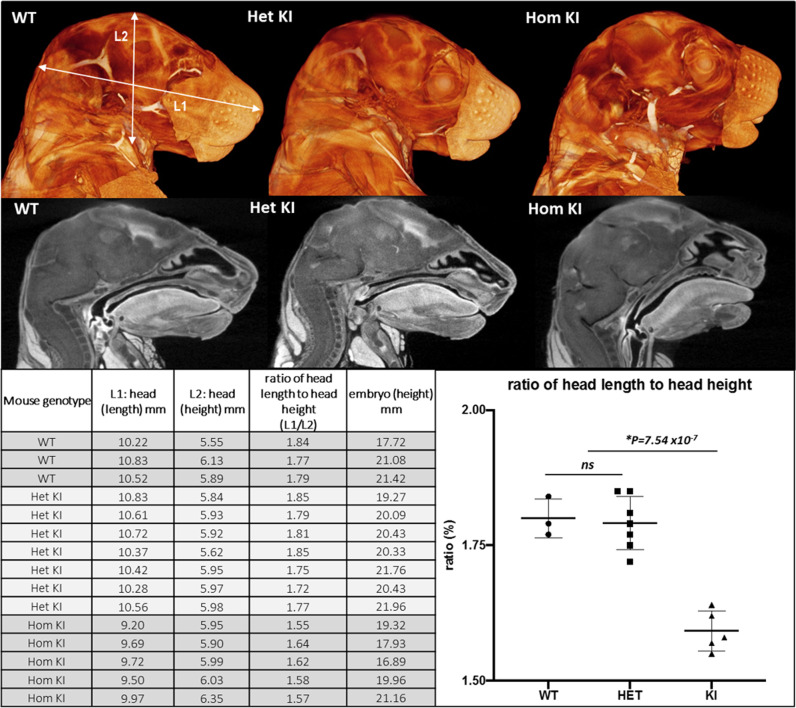
Abnormal skull shape and reduced snout length in homozygous (G682R) KI embryos. Phenotyping of *Col27a1* wild-type (WT), heterozygous *Col27a1*^*G682R/+*^, and homozygous *Col27a1*^*G682R/G682R*^ KI embryos at E18.5 with microcomputed tomography imaging. Skull shape in the homozygous mutant mice is rounder with a significantly shorter snout as compared with wild-type and heterozygous KI embryos. Abnormal skull shape and snout length were quantified as the ratio between head length (L1) over head height (L2), difference between the distribution of head ratios was evaluated between wild-type and heterozygous KI embryos which appear normal and homozygous KI embryos.

Initial generation of this engineered mouse line was performed on a C57BL/6N (Black 6) genetic background, however multiple rounds of breeding failed to deliver any surviving homozygous animals at time of genotyping. Consequently, heterozygous KI mice were bred to 129S6/SvEvTac wild-type (WT) mice to outcross and generate a mixed background line. Matings of heterozygous animals with this hybrid genetic background were able to produce viable, albeit only a few, homozygous KI animals (*N* = 7) that survived past weaning age. Genotyping of three litters at postnatal day 1 (*N* = 23 pups) revealed expected Mendelian ratios for the different genotypes: 21.7% WT (*n* = 5), 52.2% heterozygous (*Col27a1*^G682R/+^; *n* = 12), and 26.1% homozygous (*Col27a1*^G682R/G682R^; *n* = 6) KI mice. However, homozygous KI mice displayed severe lethality before P7, with only a few pups surviving post weaning age. Phenotyping of homozygous KI embryos at E18.5 showed abnormal skull shape with a shortened snout, but no significant differences in embryo size or length at this stage (Supplementary Fig. 4). This was further quantified as the ratio of head length to head height, showing that snouts of homozygous KI embryos were significantly shorter (*P* = 7.54 × 10^−7^) than those of heterozygous KI or WT embryos (Fig. 3). In addition, homozygous KI embryos had abnormal lungs with poorly developed airspaces and thickened mesenchyme (data not shown).

**Fig. 4:**
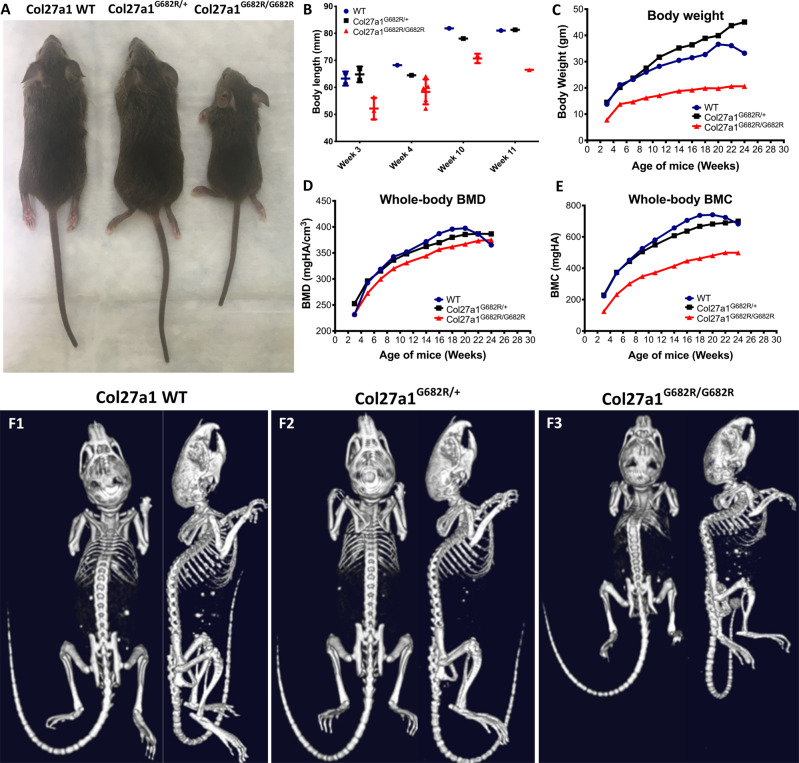
Phenotyping of *Col27a1* knockin (KI) mutant mice. **a** Homozygous *Col27a1*^G682R/G682R^ KI mice displayed significant dwarfism, heterozygous *Col27a1*^G682R/+^ KI mice were phenotypically normal and indistinguishable from wild-type littermates at 3 weeks of age. **b**–**e** Longitudinal monitoring of length, body weight, and skeletal parameters: bone mineral content (BMC) and bone mineral density (BMD) in homozygous and heterozygous KI mice versus wild-type littermates. Homozygous (*Col27a1*^G682R/G682R^) KI mice are smaller in length (**b**), display decreased body weight (**c**), and BMC (**e**) consistent with their smaller body size, but no difference in normalized BMD (**d**). **f** Dorsal and lateral µCt images of wild type (F1), heterozygous KI (F2), and homozygous KI (F3). Heterozygous KI mice do not appear different from wild-type littermates, whereas homozygous KI mice are smaller in size, display kyphosis, a shorter snout and rounded skull.

Seven homozygous KI mice, from different litters, survived past weaning age and were subjected to further evaluation for gross phenotypic and skeletal changes. We performed computerized tomography (µCT) whole-body scans of heterozygous (*Col27a1*^G682R/+^) and homozygous (*Col27a1*^G682R/G682R^) KI mice and WT littermates to evaluate for gross skeletal abnormalities and reduced body length as a surrogate for the short stature phenotype observed in the human subjects [27]. We measured the snout to base of the tail (at caudal vertebra 4, CA4) distance to evaluate differences in body length among the heterozygous, homozygous, and WT mice. Homozygous mutant mice were evidently smaller compared with WT or heterozygous littermates (Fig. 4a). Total body length was markedly decreased (Fig. 4b, f) and all the long bones were shorter. Homozygous KI mice also showed distinct craniofacial abnormalities including shorter snout and slightly rounded dome shaped skull, also observed in the embryonic stage (Fig. 3 and Supplementary Fig. 4). Analysis of the vertebral column demonstrated severe thoracic kyphosis at 3 weeks of age in all the homozygous KI mice evaluated, leading to an acute angle between the skull and cervical vertebrae resulting in inward curvature of the rib cage. Homozygous KI mice also developed varus deformity observed by µCT as a structural deformity of the hind limbs with tibial tuberosity shifting inwards resulting in the tibia being twisted towards the midline and causing the digits to point inwards when mice are in the supine position (Supplementary Fig. 5). About two thirds of the heterozygous mice evaluated also had a milder presentation of a varus deformity (Supplementary Fig. 5). Analysis of skeletal parameters revealed that homozygous mutant mice displayed lower BMC as compared with heterozygous KI and WT mice (Fig. 4d, e) consistent with their smaller size and overall reduced whole-body bone volume. Homozygous mice were half the body weight compared with WT littermates, and their growth curve remained lower throughout the monitoring period of 6 months compared with WT and heterozygous littermates. Heterozygous mice were viable and did not display any other gross skeletal abnormalities beyond the above mentioned mild varus deformity in some; skeletal parameters were indistinguishable from WT littermates. We monitored homozygous and heterozygous KI mice over a 6 month period; aging did not result in development of other gross or detectable skeletal abnormalities at a later age in heterozygous mice.

### Histological analyses of Steel syndrome mouse model and growth plate defects

**Fig. 5:**
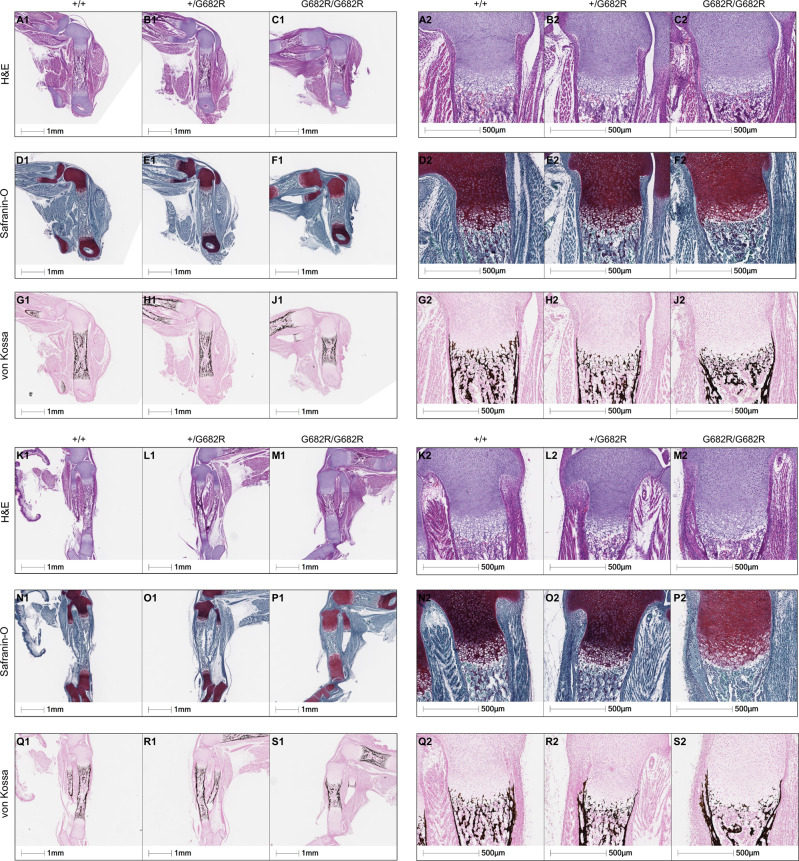
Histological analyses of long bones from *Col27a1* G682R KI mice. **a**–**j** The growth plate of the developing femur of wild-type, heterozygous *Col27a1*^*G682R/+*^, and homozygous *Col27a1*^*G682R/G682R*^ KI mice at post-natal day 1 stained with hematoxylin and eosin (A1–C1 and A2–C2 at higher magnification), safranin-O (D1–F1 and D2–F2 at higher magnification) and von Kossa (G1–J1 and G2–J2 at higher magnification). **k**–**s** The growth plate of the developing tibia of wild-type, heterozygous *Col27a1*^*G682R/+*^, and homozygous *Col27a1*^*G682R/G682R*^ KI mice at post-natal day 1 stained with hematoxylin and eosin (K1–M1 and K2–M2 at higher magnification), safranin-O (N1–P1 and N2–P2 at higher magnification) and von Kossa (Q1–S1 and Q2–S2 at higher magnification). Homozygous *Col27a1*^*G682R/G682R*^ KI mice displayed chondrodysplasia with reduced and disorganized proliferative zone and complete absence of columnar chondrocytes as compared with wild-type and heterozygous KI littermates (**c**, **f**, **j**, **m**, **p**, **s**). Although the length and overall structure of the growth plate is preserved in heterozygous KI mice as compared with wild-type littermates, some disorganization of the columnar chondrocytes can be observed (**b**, **e**, **l**, **o**) Histochemical comparison of Safranin-O and von-Kossa stains displayed no overt differences in proteoglycan accumulation and mineralization respectively between genotypes.

To further explore the Steel syndrome founder allele for potential insights into skeletal biology, and the potential mechanism(s) by which defects in *COL27A1* result in osteochondrodysplasia, we collected long bones (femurs and tibias) from heterozygous (*Col27a1*^G682R/+^) and homozygous (*Col27a1*^G682R/G682R^) KI mice, and WT littermates at P1 to perform histological analyses. Stained sections of femoral and tibial growth plates of homozygous KI mice showed loss of the normal architecture of the proliferative zone with absence and disorganization of columnar chondrocytes (Fig. 5a–c and k–m, Supplementary Figs. 6 and 7). We observed a qualitative increase in PCNA-positive cells in the proliferative zone of the heterozygous and homozygous KI mice as compared with WT littermates (Supplementary Fig. 6). Histochemical comparison of Safranin-O and von-Kossa stains displayed no overt differences in proteoglycan accumulation and mineralization in homozygous KI mice versus heterozygous KI and WT mice (Fig. 5d–j and n–s). Similarly, immunohistochemical analysis demonstrated no overt differences in Collagen II and Collagen X staining in the growth plate region (Supplementary Fig. 7). Interestingly, while the length and gross morphology of the long bones in heterozygous KI mice is not different from WT littermates, histological analyses of the growth plates in these mice revealed an intermediate phenotype, where the length of the proliferative zone of the growth plate is maintained but some disorganization in the columnar arrangement of chondrocytes can be observed (Fig. 5, B1, B2, L1, L2, and Supplementary Figs. 6 and 7).

### In vitro functional characterization of disease associated missense variants in COL27A1

To elucidate the potential molecular mechanism underlying the disease trait and clinically observed phenotypes due to point mutations in *COL27A1*, we evaluated whether the two observed glycine substitutions (p.Gly697Arg and p.Gly895Arg) within the triple-helical domain of COL27A1 interfered with the biosynthesis of the protein in vitro (Supplementary Methods). To this end, we overexpressed GFP-tagged WT, G697R, or G895R human *COL27A1* constructs in W20 or ATDC5 cells, a mouse chondrogenic cell line that serves as an appropriate in vitro model for chondrocyte differentiation [28], and evaluated ER retention by co-localizing with an ER-retained ER-Crimson fluorophore. Our experiments showed modest ER-retention of the mutant proteins as compared with the WT in both W20 and ATDC5 cells (Supplementary Fig. 8A, B), suggesting that potential disruption of the triple helical collagen domain of COL27A1 may lead to abnormal folding and secondary structure. However, we did not specifically test for ER-stress or upregulation of the unfolded protein response. We tested for defects in chondrogenic differentiation of ATDC5 cells in vitro when overexpressing the mutant constructs versus WT. We used alcian blue staining to visualize proteoglycan accumulation at different multiplicity of infection (MOIs) (10, 20, 50, and 100) for the three constructs; however, we did not observe any major or significant differences between the mutations and WT *COL27A1* overexpressing cells (Supplementary Fig. 8C).

It is possible that the observed ER-retention is a result of the supra-physiological levels of the overexpressed mutated proteins within the cell as chondrogenic differentiation of ATDC5 overexpressing G697R or G895R mutant proteins was unremarkable as compared with WT. In order to exclude any potential artifacts due to overexpression, we isolated primary chondrocytes from postnatal day 1 (P1) heterozygous (*Col27a1*^G682R/+^) and homozygous (*Col27a1*^G682R/G682R^) KI mice and WT littermates. After 2 weeks in culture, we performed alcian blue staining to test for chondrogenic defects among the different genotypes. Consistent with our previous observation in ATDC5 cells, we did not observe significant differences in chondrogenesis between genotypes as evaluated by proteoglycan alcian blue staining (Supplementary Fig. 9). These experiments led us to conclude that *Col27a1* mutant chondrocytes are able to differentiate and secrete collagen into the extracellular matrix (ECM) and that the mechanism of disease in STLS and related osteochondrodysplasias is likely not cell-autonomous.

## Discussion

Fibrillar collagens are one of the most well-described, abundant, and relevant protein families that form major structural components of extracellular matrices in vertebrates. Collagen XXVII, i.e. COL27A1 the product of the human and murine *COL27A1/Col27A1* gene, is a type C fibrillary collagen that is developmentally regulated and expressed in a variety of tissues including the developing dermis, eye, otic capsule, bronchial and lung epithelium, and major arteries in the heart during early embryonic development (mouse E14.5); however at later stages in development and throughout adulthood it is most prominently and exclusively expressed in cartilage [29, 30]. The protein has been identified in primary ossification centers, in the proliferative chondrocytes of the epiphyseal growth plate, and in the pericellular matrix surrounding the hypertrophic chondrocytes at the transition zone from cartilage to bone [29–33]. It has been proposed from a developmental biology standpoint that this uncharacteristic collagen plays an important role in the cartilage modeling phase of endochondral bone formation where cartilage mineralization and apoptosis of hypertrophic chondrocytes occurs with invasion of blood vessels and osteogenic cells, deposition of collagen I and bone formation, therefore playing an important role in skeletogenesis [29].

We previously reported the association of a rare *COL27A1* missense variant, c.2089G>C (p.Gly697Arg), causing Steel syndrome when in homozygosis in three members of a single family; female/male sibs and a female cousin [4]. Due to the absence of reported consanguinity, which was further verified genetically by the lack of sharing of large segments of homozygosity beyond the region around the *COL27A1* gene delineating a common haplotype harboring the c.2089G>C (p.Gly697Arg) variant, at the time we proposed that this variant was the molecular cause of the disease and that it was likely a founder allele in individuals of Puerto Rican ancestry. Since then, we and others [5, 23] have confirmed that homozygosity for this missense variant is indeed the molecular defect that causes the skeletal abnormalities observed in STLS.

In this report, we now show that five of the original STLS patients reported by Steel et al. [1] are homozygous for this same c.2089G>C (p.Gly697Arg) variant and that it segregates in these Puerto Rican families according to Mendelian expectations for an AR disease trait. In addition, we report five more individuals, including four adults, of Puerto Rican descent and with clinical features consistent with STLS. As expected, these individuals were also found to be homozygous for the STLS variant. In aggregate, a total of 47 patients have been described, including the ones documented in this report, with clinical findings corresponding to “The Puerto Rican syndrome” that Dr Steel reported 50 years ago. Of these, 20 have been molecularly confirmed and reported to be homozygous for the c.2089G>C (p.Gly697Arg) variant in *COL27A1*, all of Puerto Rican ancestry, confirming our founder allele hypothesis for this disorder [4]. The reported cases also document the specificity of the genotype–phenotype association consistent with a relatively uniform clinical phenotype characterized primarily by bilateral congenital hip dysplasia (100%), short stature (95%), scoliosis (85%), carpal coalitions (77%), radial head dislocations (65%), foot deformities, and vertebral anomalies (~50–65%) (Tables 1 and 2).

We now also report three unrelated probands from Turkey with homozygous novel pathogenic variants (c.2683G>A:p.(Gly895Arg), c.4976_4980delGAGGA:p.(Gly1660Aspfs*3), and c.63-4_69del:p.(Gly22Serfs*6)]) in *COL27A1*. These three individuals, in addition to recent case reports of unrelated patients with skeletal dysplasia within the spectrum of STLS, were found to have biallelic rare protein altering variants in *COL27A1* consistent with an AR disease trait [7–11] (Fig. 1f). These findings expand on the phenotypic and variation spectrum of function altering alleles in this gene enabling the characterization of an allelic series (Table 2 and Fig. 1). Given the phenotypic overlap but increased severity and additional features not observed or reported to date in the majority of the Puerto Rican STLS patients, such as congenital or early onset hearing loss, speech and developmental delay, and novel radiographic features including tall vertebral bodies, long vertebral pedicles, and shallow glenoid fossae (Fig. 1), we propose these to represent an allelic series of COL27opathies with potentially a broader phenotype spectrum due to biallelic function altering variants in *COL27A1* and allowing a more complete clinical synopsis of this locus. In contrast to the Puerto Rican founder allele for STLS, the disease associated alleles identified in the Turkish population along with the reported protein altering variants in non-Puerto Rican cases were all distinct variants. This observation is more consistent with a Clan Genomics hypothesis [12] of recently arisen rare alleles that are more likely to come together due to IBD homozygosity driven by consanguinity rather than genetic drift due to population bottlenecks and founder mutational events. The high percentage of genome-wide AOH due to recent IBD homozygosity observed in the Turkish subjects is also consistent with this interpretation.

Based on the disease-causing variation spectrum observed in humans, it appears that some of the clinical variability among patients with different variants may be due to the functional effect of the variant and the extent of perturbation to the translated protein (Fig. 1a). The identified protein altering amino acid substitutions (p.Gly697Arg, p.Gly895Arg, and p.Gly904Arg) affect the triple helical collagen domain of COL27A1. Interestingly, similar mutations in other collagen types may act as dominant alleles through dominant negative or antimorphic effects. Missense variants affecting this domain in COL27A1 are indeed function altering as demonstrated by STLS and the Turkish case (BAB5133) reported here, but the severity of the phenotype might be dependent on the location of the alteration within the triple-helical domain. Our observations are consistent with previous experiments from Plumb et al. [29] where no significant engorgement of the ER was observed in chondrocytes of an in-frame deletion mutant mouse model, suggesting that protein misfolding and ER retention [34, 35] were unlikely to play a significant role in the pathology associated with abnormal COL27A1. Furthermore, a different Glycine substitution (p.Gly1516Cys) towards the end of the triple-helical domain of COL27A1 showed little phenotypic effect in this same study, with some disordering of the proliferative zone of growth plates in homozygous animals [29]. In aggregate, these data support the hypothesis that members of the type C clade of collagens, of which COL27A1 is a member of along with COL24A1, may be more tolerant, in terms of folding and secretion, to disruptions of the Gly-X-Y motif in their triple-helical domain [29–31]. In addition, collagen XXVII homotrimerizes into thin fibrils in the ECM independently from the thicker fibrils formed by classical (type A and B) fibrillar collagens possibly allowing to better accommodate the mutant collagen trimers [30, 32]. These observations further support the observed recessive inheritance of COL27opathies associated pathogenic alleles as compared with the usual dominant inheritance pattern observed for mutations in other classical collagens and the hypothesis that the phenotypic effects of mutations in the triple-helical domain may vary depending on the location within this.

The two additional variants reported here are predicted to introduce frameshift or splice changes that result in the introduction of early termination codons that lead to nonsense mediated processing and clearance of the mutant transcripts, effectively resulting in loss-of-function (LoF) alleles with consequent loss of the COL27A1 protein. These two Turkish probands, in addition to the cases reported by Kotabagi et al. [8] and Pölsler et al. [11] are interesting as they reveal distinctions in the role and importance of collagen XXVII in human versus other mammalian skeletal systems. A previously reported mouse model of *Col27a1* deficiency exhibited severe skeletal abnormalities with chondrodysplasia and perinatal death due to lung defects [29]. In contrast to the previously reported mouse models of *Col27a1* mutation that aimed to assess function of this collagen gene without a human phenotype reference, our engineered mouse model contains the orthologous variant equivalent to the most prevalent function altering variant and human disorder associated with this gene. Our STLS KI animal model (*Col27a1*^G682R/G682R^) recapitulates the short stature and some of the skeletal abnormalities observed in the human subjects, however most homozygous KI mice die perinatally, presumably due to respiratory insufficiency consistent with previous *Col27a1* mouse models [29]. Complete absence and hypomorphic alleles in the mouse *Col27a1* gene result in neonatal lethality in addition to severe skeletal abnormalities; however markedly reduced and apparent absence of COL27A1 protein in humans appears to be viable albeit with the reported skeletal abnormalities in the spectrum of STLS and related COL27opathies. Interestingly, STLS patients do not appear to have respiratory problems or lung abnormalities.

Based on our in vitro experiments and observations in our STLS in vivo mouse model, it appears that disease associated alterations in *COL27A1* do not prevent chondrocytes from differentiating properly. However abnormal or absent collagen XXVII protein in the ECM results in collapse of the proliferative zone and disorganization of the cartilaginous zones of the growth plate preventing normal endochondral bone growth possibly through impaired interactions with other ECM proteins or matrix-sequestered ligands. Our observations further support the model of collagen XXVII as an important scaffold for cartilage mineralization and population of other cell types such as endothelial and osteogenic cells during skeletal development. Which ligands or ECM factors are being altered by the absence or disruption of collagen XXVII remain a subject of future research; however, our STLS model provides a biologically relevant platform to study these further mechanistic questions regarding COL27opathies with direct human biology translation.

Mouse *Col27a1* was found to be expressed in the cartilaginous structures associated with inner ear development and cochlear epithelium [32]. This observation may be of relevance considering the associated hearing loss phenotypes observed in individuals with severe and LoF variants in *COL27A1*. Interestingly, in family HOU2809 composed of three affected siblings with STLS but molecularly diagnosed and clinically evaluated as adults (ages 33, 39, and 48 years), all three affected individuals presented with bilateral hearing loss with onset after 30 years of age. Similarly, patient GHS01 identified in the DiscovEHR cohort also developed bilateral sensorineural hearing loss at 38 years of age. While hearing loss had not been reported in individuals with STLS, most of the reported patients are children and therefore it is plausible that in Puerto Rican STLS patients due to homozygosity for the missense c.2089G>C (p.Gly697Arg) variant, hearing loss may appear later in life versus early-onset hearing loss observed in individuals with more severe variants in *COL27A1*. Our data on the clinical characterization of adult STLS patients supports this conclusion.

Belbin et al. suggested that the Puerto Rican c.2089G>C (p.Gly697Arg) Steel syndrome variant has phenotypic consequences in both heterozygous and homozygous individuals [5]. The authors confirmed a clinical diagnosis based on EHR diagnostic codes consistent with STLS for only the five identified homozygous individuals in their Puerto Rican cohort (*N* = 115). However as reported, phenome-wide association analyses of ICD-9 medical billing codes in the EHR of c.2089G>C (p.Gly697Arg) heterozygous carrier individuals failed to identify significantly associated phenotypes, except for one association with vertebral osteomyelitis driven by three heterozygous individuals. As stated by the authors, manual examination of EHR data for a subset of heterozygous carriers (*N* = 34), using the same clinical criteria that was used for evaluation of the five homozygous individuals, found no evidence of short stature or congenital hip dislocation consistent with the major observed phenotypes in STLS. Through this manual examination, the authors observed enrichment of joint and spine degradation diagnosis codes in the EHR data, including moderate-to-severe spinal degeneration and cervical stenosis and increased rates, albeit not statistically significant, of scoliosis, arthritis, and lumbar spine degradation in the evaluated c.2089G>C (p.Gly697Arg) heterozygous carriers.

Similar interrogation of the EHR information of 28 heterozygous carriers of the STLS c.2089G>C (p.Gly697Arg) variant in the DiscovEHR cohort showed occurrences of diagnosis codes for degenerative joint disease and joint pain, and reported pain in the neck, back and limbs, and some instances of scoliosis and osteoarthritis. However, none of these were statistically significant or consistent across all heterozygous carriers of the STLS variant. Our observations, along with those reported by Belbin et al. are in line with previous observations of subclinical phenotypes in heterozygous carriers for recessive disorders and late onset of related disease with milder presentations due to a sensitized genetic background and life-long exposures in heterozygotes [36, 37]. Interestingly, initial findings from a Chinese cohort of subjects with scoliosis have identified enrichment for heterozygous deleterious rare variants in *COL27A1* (unpublished data). In addition, we observed mild varus deformity and histological differences in the growth plate of heterozygous KI mice for our STLS model with moderate disorganization of the proliferative zone and the columnar arrangement of chondrocytes. Despite this, the length of the growth plate and overall size and gross morphology of heterozygote KI animals was comparable with WT mice. These observations strongly suggest that whilst the vertical growth is unaffected, the overall skeletal structure in individuals heterozygous for the c.2089G>C (p.Gly697Arg) variant may be sensitized and more prone to degeneration with age and environmental insults and exposures. Altogether, these observations might be more consistent with the *COL27A1* locus contributing to complex osteopathological traits in sensitized heterozygous individuals rather than reflecting an autosomal dominant disease trait.

Of note, the majority of patients with STLS and associated osteochondrodysplasias, COL27opathies, are children. Few adults have been well-phenotyped and followed-up in their clinical progression with the disease, therefore worsening of some of the phenotypes such as vertebral compression, scoliosis, and hearing loss may potentially appear with age. This age-dependent penetrance of some clinical phenotypes is illustrated by the three adult STLS individuals from pedigree HOU2809 and individual GHS01 that we report herein who developed bilateral hearing loss of adult onset, and progressive pain and walking difficulties. Similarly, *COL27A1* pathogenic variants may confer complex trait susceptibility to scoliosis, vertebral and joint degeneration, and hearing loss as homozygous and heterozygous carriers age and given additional environmental factors such as lifestyle including weight and exercise routines or noise exposures.

We have demonstrated apodictically that Steel syndrome is an AR disorder clinically characterized by congenital bilateral hip dislocation, short stature, scoliosis, radial head dislocation, carpal coalitions, and foot deformities; and molecularly defined by homozygosity for the c.2089G>C (p.Gly697Arg) variant in *COL27A1*. The reported allelic series in *COL27A1* provides further insights into disease biology and genetic contributions to clinical phenotypes in these COL27opathies. Our in vivo model offers additional evidence for the role of collagen XXVII as an important element of ECM composition and growth plate organization for endochondral bone formation during vertebrate skeletogenesis.

It has been estimated that the c.2089G>C (p.Gly697Arg) variant exists at a minor allele frequency of 1.9% in the Puerto Rican population [5]. This allele frequency has strong implications for prevalence estimates of Steel syndrome but furthermore for the accurate diagnosis and appropriate orthopedic management of these individuals. Retrospective evaluation of STLS patients concluded that the hip dislocation is refractory to surgery and that instead of improving quality of life, surgical intervention is largely contraindicated for these patients as chronic pain and hip subluxation with acetabular dysplasia are major iatrogenic outcomes of such surgical intervention [2]. Implementation of molecular genetics for the accurate diagnosis of patients with rare diseases such as STLS or in newborn screen paradigms for populations at higher risk can prevent unnecessary treatment interventions that could potentially lead to life-long distress and disability.

Steel syndrome and associated COL27opathies due to function altering variants in the gene *COL27A1* demonstrate how rare recessive variants can come together and account for disease burden in specific populations by autozygosity either through genetic drift of founder alleles in culturally [25, 26] or geographically isolated populations such as in Puerto Rico, or consistent with the Clan Genomics hypothesis [12], through rapid intergenerational IBD homozygosity due to increased consanguinity of parents or “clan members” of affected probands. In addition, the observation that some of these rare variants can contribute in heterozygosity [36] or in combination with common polymorphisms [38] to more common complex disorders or to late-onset related clinical presentations [37] due to life-long exposures in individuals with a sensitized genetic background, gene–environment interactions, further supports the importance of recent rare variation arising in the “clan” as an important contributor to disease susceptibility, versus common variation across multiple populations, and the continuum that connects rare and common diseases.

## Supplementary information

Supplementary Material

## References

[1] Steel HH, Piston RW, Clancy M, Betz RR (1993). A syndrome of dislocated hips and radial heads, carpal coalition, and short stature in Puerto Rican children. J Bone Jt Surg Am..

[2] Flynn JM, Ramirez N, Beta R, Mulcahey MJ, Pino F, Herrera-Soto JA (2010). Steel syndrome: dislocated hips and radial heads, carpal coalition, scoliosis, short stature, and characteristic facial features. Pediatr Orthop.

[3] Steel HH. The Puerto Rican syndrome, Read at the Annual Meeting of Shrine Surgeons. San Francisco; 1973.

[4] Gonzaga-Jauregui C, Gamble CN, Yuan B, Penney S, Jhangiani S, Muzny DM (2015). Mutations in COL27A1 cause Steel syndrome and suggest a founder mutation effect in the Puerto Rican population. Eur J Hum Genet..

[5] Belbin GM, Odgis J, Sorokin EP, Yee MC, Kohli S, Glicksberg BS (2017). Genetic identification of a common collagen disease in puerto ricans via identity-by-descent mapping in a health system. Elife.

[6] Moreno-Estrada A, Gravel S, Zakharia F, McCauley JL, Byrnes JK, Gignoux CR (2013). Reconstructing the population genetic history of the Caribbean. PLoS Genet..

[7] Gariballa N, Ben-Mahmoud A, Komara M, Al-Shamsi AM, John A, Ali BR (2017). A novel aberrant splice site mutation in COL27A1 is responsible for Steel syndrome and extension of the phenotype to include hearing loss. Am J Med Genet A..

[8] Kotabagi S, Shah H, Shukla A, Girisha KM (2017). Second family provides further evidence for causation of Steel syndrome by biallelic mutations in COL27A1. Clin Genet..

[9] Maddirevula S, Alzahrani F, Al-Owain M, Al Muhaizea MA, Kayyali HR, AlHashem A (2019). Autozygome and high throughput confirmation of disease genes candidacy. Genet Med..

[10] Thuresson AC, Soussi Zander C, Zhao JJ, Halvardson J, Maqbool K, Månsson E (2019). Whole genome sequencing of consanguineous families reveals novel pathogenic variants in intellectual disability. Clin Genet..

[11] Pölsler L, Schatz UA, Simma B, Zschocke J, Rudnik-Schöneborn S (2020). A Syrian patient with Steel syndrome due to compound heterozygous COL27A1 mutations with colobomata of the eye. Am J Med Genet A..

[12] Lupski JR, Belmont JW, Boerwinkle E, Gibbs RA (2011). Clan genomics and the complex architecture of human disease. Cell..

[13] Lupski JR, Gonzaga-Jauregui C, Yang Y, Bainbridge MN, Jhangiani S, Buhay CJ (2013). Exome sequencing resolves apparent incidental findings and reveals further complexity of SH3TC2 variant alleles causing Charcot-Marie-Tooth neuropathy. Genome Med..

[14] Dewey FE, Murray MF, Overton JD, Habegger L, Leader JB, Fetterolf SN (2016). Distribution and clinical impact of functional variants in 50,726 whole-exome sequences from the DiscovEHR study. Science.

[15] Karaca E, Posey JE, Coban Akdemir Z, Pehlivan D, Harel T, Jhangiani SN (2018). Phenotypic expansion illuminates multilocus pathogenic variation. Genet Med..

[16] Coban-Akdemir Z, White JJ, Song X, Jhangiani SN, Fatih JM, Gambin T (2018). Identifying genes whose mutant transcripts cause dominant disease traits by potential gain-of-function alleles. Am J Hum Genet.

[17] Manichaikul A, Mychaleckyj JC, Rich SS, Daly K, Sale M, Chen WM (2010). Robust relationship inference in genome-wide association studies. Bioinformatics..

[18] Valenzuela DM, Murphy AJ, Frendewey D, Gale NW, Economides AN, Auerbach W (2003). High-throughput engineering of the mouse genome coupled with high-resolution expression analysis. Nat Biotechnol..

[19] Poueymirou WT, Auerbach W, Frendewey D, Hickey JF, Escaravage JM, Esau L (2007). F0 generation mice fully derived from gene-targeted embryonic stem cells allowing immediate phenotypic analyses. Nat Biotechnol..

[20] Wong MD, Dorr AE, Walls JR, Lerch JP, Henkelman RM (2012). A novel 3D mouse embryo atlas based on micro-CT. Development..

[21] Das NM, Hatsell S, Nannuru K, Huang L, Wen X, Wang L (2016). In vivo quantitative microcomputed tomographic analysis of vasculature and organs in a normal and diseased mouse model. PLoS One..

[22] Lewis RA, Shroyer NF, Singh N, Allikmets R, Hutchinson A, Li Y (1999). Genotype/phenotype analysis of a photoreceptor-specific ATP-binding cassette transporter gene, ABCR, in Stargardt disease. Am J Hum Genet.

[23] Amlie-Wolf L, Moyer-Harasink S, Carr AM, Giampietro P, Schneider A, Simon M (2020). Three new patients with Steel syndrome and a Puerto Rican specific COL27A1 mutation. Am J Med Genet A..

[24] Karaca E, Harel T, Pehlivan D, Jhangiani SN, Gambin T, Coban Akdemir Z (2015). Genes that affect brain structure and function identified by rare variant analyses of mendelian neurologic disease. Neuron..

[25] Morimoto M, Waller-Evans H, Ammous Z, Song X, Strauss KA, Pehlivan D (2018). Bi-allelic CCDC47 variants cause a disorder characterized by woolly hair, liver dysfunction, dysmorphic features, and global developmental delay. Am J Hum Genet.

[26] Williams KB, Brigatti KW, Puffenberger EG, Gonzaga-Jauregui C, Griffin LB, Martinez ED (2019). Homozygosity for a mutation affecting the catalytic domain of tyrosyl-tRNA synthetase (YARS) causes multisystem disease. Hum Mol Genet.

[27] Tan TY, Gonzaga-Jauregui C, Bhoj EJ, Strauss KA, Brigatti K, Puffenberger E (2017). Monoallelic *BMP2* variants predicted to result in haploinsufficiency cause craniofacial, skeletal, and cardiac features overlapping those of 20p12 deletions. Am J Hum Genet.

[28] Yao Y, Wang Y (2013). ATDC5: an excellent in vitro model cell line for skeletal development. J Cell Biochem.

[29] Plumb DA, Ferrara L, Torbica T, Knowles L, Mironov A, Kadler KE (2011). Collagen XXVII organises the pericellular matrix in the growth plate. PLoS One.

[30] Pace JM, Corrado M, Missero C, Byers PH (2003). Identification, characterization and expression analysis of a new fibrillar collagen gene, COL27A1. Matrix Biol..

[31] Boot-Handford RP, Tuckwell DS, Plumb DA, Rock CF, Poulsom R (2003). A novel and highly conserved collagen (pro(alpha)1(XXVII)) with a unique expression pattern and unusual molecular characteristics establishes a new clade within the vertebrate fibrillar collagen family. J Biol Chem.

[32] Plumb DA, Dhir V, Mironov A, Ferrara L, Poulsom R, Kadler KE (2007). Collagen XXVII is developmentally regulated and forms thin fibrillar structures distinct from those of classical vertebrate fibrillar collagens. J Biol Chem.

[33] Hjorten R, Hansen U, Underwood RA, Telfer HE, Fernandes RJ, Krakow D (2007). Type XXVII collagen at the transition of cartilage to bone during skeletogenesis. Bone..

[34] Khajavi M, Inoue K, Wiszniewski W, Ohyama T, Snipes GJ, Lupski JR (2005). Curcumin treatment abrogates endoplasmic reticulum retention and aggregation-induced apoptosis associated with neuropathy-causing myelin protein zero-truncating mutants. Am J Hum Genet.

[35] Khajavi M, Lupski JR (2008). Balancing between adaptive and maladaptive cellular stress responses in peripheral neuropathy. Neuron..

[36] Lupski JR, Reid JG, Gonzaga-Jauregui C, Rio Deiros D, Chen DC, Nazareth L (2010). Whole-genome sequencing in a patient with Charcot-Marie-Tooth neuropathy. N Engl J Med.

[37] Allikmets R, Shroyer NF, Singh N, Seddon JM, Lewis RA, Bernstein PS (1997). Mutation of the Stargardt disease gene (ABCR) in age-related macular degeneration. Science..

[38] Wu N, Ming X, Xiao J, Wu Z, Chen X, Shinawi M (2015). TBX6 null variants and a common hypomorphic allele in congenital scoliosis. N Engl J Med.

