# Supplementary material for: Functional biology of the Steel syndrome founder allele and evidence for clan genomics derivation of COL27A1 pathogenic alleles worldwide
Source: Eur J Hum Genet. 2020 May 6;28(9):1243–64. doi: 10.1038/s41431-020-0632-x (PMC7608441; doi:10.1038/s41431-020-0632-x)
Supplement: Supplementary file 1 — Supplementary Material [file 41431_2020_632_MOESM1_ESM.pdf]

**Functional biology of the Steel syndrome founder allele and evidence for clan genomics derivation of *COL27A1* pathogenic alleles worldwide**

**Supplementary Information and Figures**

***Supplementary Clinical Information***

**Proband BAB7802 (Family HOU2809).** An affected 39 year old female proband (BAB7802, Table 1) referred for a suspected clinical diagnosis of STLS, presented with short stature (1.47m, -2.46 SD) and bilateral congenital hip dysplasia; joint instability with numerous joint dislocations involving the knees, elbows, and toes; history of motor delay; hearing loss that developed in the $4^{th}$ decade of life; and difficulty with ambulation requiring a walker. She has severe scoliosis with lumbar lordosis, arthritis of the hip, knees, wrists and spine, and describes progressive fatigue, joint pain, and disability over the last decade. Family history is positive for two similarly affected adult siblings with short stature, congenital hip and knee dislocations, scoliosis, and hearing loss (Supplementary Information). Her affected sister, BAB9002, is a 48 year old female with a similar history of congenital bilateral hip dislocation and foot deformity characterized by displacement of the cuneiform bone and *pes planus*. Medical history illustrates a similar progression of disease, including joint laxity with recurrent joint dislocations, scoliosis with lumbar lordosis, myopia, and adult-onset bilateral hearing loss. Additional medical history is notable for hypothyroidism, Chiari malformation, and a tethered cord. Her adult height is 1.40m (-3.58 SD). The affected brother, BAB9010, is a 33 year old male with a similar history of congenital bilateral hip dislocation and

foot deformity, now with externally rotated lower extremities. His medical history also includes a similar progression of disease, including joint laxity with recurrent joint dislocations, scoliosis with lumbar lordosis, myopia, and adult-onset bilateral hearing loss. He was previously given a clinical diagnosis of Ehlers-Danlos arthrochalasia type. His adult height is 1.57m (-2.75 SD). The family is of Puerto Rican ancestry with no reported consanguinity and additionally composed of unaffected parents of normal stature (father 1.63m [-1.93 SD] and mother 1.60m [-0.51 SD]) and two other unaffected siblings of normal height. Whole-exome sequencing and targeted mutation testing in all affected siblings confirmed a molecular diagnosis of STLS due to homozygosity for the c.2089G>C (p.Gly697Arg) variant in *COL27A1*.

**Proband BAB5133 (Family HOU2031).** The proband was born to first cousin healthy parents at term by vaginal delivery with no known history of prenatal insult and intrauterine developmental delay. The parents had three *in utero* pregnancy losses of unknown etiology. After birth, a single umbilical cord, cord entanglement, and hyperbilirubinemia were noted. He suffered from frequent respiratory and ear infections during the neonatal period. The first examination for dysmorphic features at age 1 year and 3 months revealed wooly sparse hair, prominent forehead, short upturned nose, long philtrum, torticollis, rhizomelia of the upper limbs, bowing of femurs, rocker bottom feet, *pes planovalgus*, and joint laxity. He was short, had mild developmental delay and unilateral conductive deafness due to recurrent infections. He had surgical intervention due to a large ventricular septal defect and pulmonary hypertension. Routine metabolic tests, calcium, ALP, PTH, thyroid function tests, urine and blood glycosaminoglycan levels were at normal ranges. X-rays showed bowing bent femurs, delayed epiphyseal ossification and hypoplastic clavicles and prominent metaphyseal irregularity. Lumbar kyphosis was also noted.

X-rays of lateral spine and forearms at 5 months of age revealed tall vertebral bodies, long vertebral pedicles and possibly dislocated radial heads. Robinow syndrome and campomelic dysplasia were the initial diagnoses considered based on the physical examination. However, *SOX9* analysis and exome sequencing showed no rare variants in the known genes associated with these disorders. WES analysis of the proband identified a homozygous variant in exon 19 of *COL27A1* [hg19:g.chr9:116999951(G>A); c.2683G>A; p.Gly895Arg].

***Supplementary Methods***

***Targeting vector and allele modification to generate Col27a1 (p.Gly682Arg) knock-in mutant mice.*** To model the human p.Gly697Arg variant, a guanine-to-cytidine mutation, resulting in a p.Gly682Arg substitution (orthologous to the human variant), was introduced in exon 7 of 61 (exonID: ENSMUSE00001215121 [build GRCm38]) of the mouse gene. Briefly, a DNA construct (GenScript) containing *Col27a1* exon 7 and surrounding intronic sequences was synthesized to include the desired G>C mutation, as well as two small intronic deletions (away from known regulatory sites) to accommodate loss-of-allele (LOA) TaqMan assays TU and TD. An intermediate donor vector was created by cloning a floxed hygromycin resistance self-deleting cassette (SDC) into restriction sites introduced to intron 7. The donor plasmid was linearized by *in vitro* cleavage with S. pyogenes Cas9/gRNA complexes, outside the intronic deletions and cassette. A murine BAC (RP23-448J19) containing *Col27a1* was separately cleaved with two additional guides designed to create complementary overhangs with the donor vector. The donor and the BAC were combined together using Gibson assembly to create the final targeting vector. After

extensive QC and linearization, the final targeting vector was electroporated into C57BL/6N mouse embryonic stem cells and selected via hygromycin resistance in the self-deleting cassette inserted in the downstream intron. Mice carrying the introduced mutation were confirmed by Sanger dideoxynucleotide sequencing genotyping.

***Plasmid construction for in vitro evaluation of COL27A1 mutations.*** Human *COL27A1* cDNA was obtained (Origene #RC214474) and two point mutations (p.Gly697Arg [G697R] and p.Gly895Arg [G895R]) were introduced through site directed mutagenesis (SDM) using the Agilent QuikChange Lightning kit (Agilent #210518), according to the manufacturer's protocol. SDM oligos for introducing the G697R (5'-TGACATGGGCTTGCCTCGGCTCTCCG-3'; 5'-CGGAGAGCCGAGGCAAGCCCATGTCA-3') and G895R (5'-ATGGATCCTTTGTCTCTGACTTTGCCCAGAGGC-3'; 5'-GCCTCTGGGCAAAGTCAGAGACAAAGGATCCAT-3') variants were used for mutagenesis.

COL27A1-G697R, COL27A1-G895R and wild-type (COL27A1-WT) constructs were subcloned into vectors encoding a C-terminal mGFP tag (pCMV6-AC-mGFP, Origene #PS100040). Final constructs were confirmed by Sanger dideoxynucleotide sequencing.

***In vitro cell culture.*** Low passage W-20-17 [W-20 clone 17] (ATCC® CRL- 2623TM) and ATDC5 cells (Yao et al, 2013) were maintained in DMEM (with 4mM L-glutamine, 1.5g/L sodium bicarbonate, 4.5g/L glucose, 10% heat-inactivated FBS, and 1% penicillin-streptomycin) and DMEM:Ham's F12 (with 2mM glutamine, 5% FBS) at 5% $CO_2$ and 37 °C, respectively. W20 cells were plated at 40000cells/cm2 on glass bottom, poly-l-lysine coated Ibidi chamber slides (Cat # 80427). Cells

were transfected with ER-E2-Crimson (pEF.myc.ER-E2-Crimson, Addgene # 38770) along with pUC19, pCMV6-AC-mGFP-COL27A1-WT, pCMV6-AC-mGFP-COL27A1-G697R or pCMV6-AC-mGFP-COL27A1-G895R using X-tremeGENE HP DNA transfection agent (Roche #06 366 244 001) at 1:4 (DNA:transfection agent) ratio. Media was replaced a day after transfection, and the following day 50 μg/ml of L-ascorbic acid was added to the media. A day later cells were fixed with freshly prepared 4% paraformaldehyde in DPBS with calcium and magnesium for 15 minutes at room temperature, cells were either imaged directly or processed for immunostaining using anti-GFP antibody (CST #2956, 1:100 dilution) as per the manufacturers recommendation. Images were acquired on a Zeiss LSM 880 laser scanning microscope. Similarly, ATDC5 cells were plated at 40000cells/cm2 on poly-l-lysine coated, glass bottom Ibidi slides and co-transduced using adenoviral particles encoding ER-RFP [multiplicity of infection (MOI) 20] along with COL27A1-WT, COL27A1-G697R or COL27A1-G895R at MOI ranging from 10-100 per cell. The cells were then fixed with 4% paraformaldehyde in DPBS with calcium and magnesium for 10 minutes at room temperature and then imaged using a Zeiss LSM 880 microscope.

***Cell transduction and histochemical analyses.*** ATDC5 cells were plated at 4000 cells/well in a 12-well plate and transduced the following day with adenoviral particles encoding COL27A1-WT, COL27A1-G697R or COL27A1-G895R. The next day, they were changed into culture medium containing ITS supplement (Gibco #41400045) to induce chondrogenic differentiation as per Shukunami et al. On day 30 of chondrogenic induction, the cells were fixed in 95% methanol at room temperature for 20 minutes, stained with 1% Alcian blue 8GX in 0.1M HCl overnight, and imaged. The Alcian blue was then extracted by incubation with 6M guanidine HCl for 6 hours at

room temperature, and quantified through absorbance measurement of the resulting solutions at 630nm, using a SpectraMax M4 microplate reader.

***In vitro cell culture of mouse primary chondrocytes.*** Primary chondrocytes were collected from mouse neonates (P1) after euthanasia following approved IACUC guidelines and according to established protocols (Gosset et al, 2008). Epiphyseal cartilage from femur, tibia, and humerus was collected using a dissecting microscope. Cartilage was further dissected into 2-3 pieces per sample and digested in 5ml of 0.25% trypsin in HBSS for 1hr at 37 °C. Supernatant was discarded from sedimented samples, remaining cartilage pellets were further digested overnight at 37 °C using 10ml of 0.75mg/ml of collagenase I (Worthington Biochemical Corporation, CLS1). Upon overnight digestion, chondrocytes were dissociated from the cartilage pieces by repeated pipetting (20 times) using a glass Pasteur pipette. Dissociated cells were passed through a cell strainer (80-100µm). Cells were subsequently washed twice with 10ml growth media (10% MEM). Cells were seeded at 150000/well in a collagen-coated 24-well plate. Upon confluency, 50 µg/ml of L-ascorbic acid was added to the growth media. After two-weeks in culture, cells were fixed in 10% neutral buffered formalin for 15 minutes at room temperature. After multiple washes with double distilled water, cells were treated with 70% ethanol for 5 minutes at room-temperature. Subsequently, cells were washed with double distilled water and incubated with 5% acetic acid (pH: 1.0) for 5 minutes at room temperature. Immediately after, cells were incubated with 1% Alcian blue 8GX (SIGMA A3157) for 2 hours at room temperature. Cells were de-stained in water (5 washes, 10min/wash), followed by an overnight wash at 4 °C. Cells were imaged using an EVOS digital microscope.

**Supplementary Methods References:**


1. Yao Y, Wang Y. ATDC5: an excellent in vitro model cell line for skeletal development. J Cell Biochem. 2013 Jun; 114(6):1223-9.
2. Shukunami C, Shigeno C, Atsumi T, Ishizeki K, Suzuki F, Hiraki Y. Chondrogenic differentiation of clonal mouse embryonic cell line ATDC5 in vitro: differentiation-dependent gene expression of parathyroid hormone (PTH)/PTH-related peptide receptor. J Cell Biol. 1996; 133(2):457-68.
3. Gosset M, Berenbaum F, Thirion S, Jacques C. Primary culture and phenotyping of murine chondrocytes. Nat Protoc. 2008; 3(8):1253-60.

*Supplementary Figures*

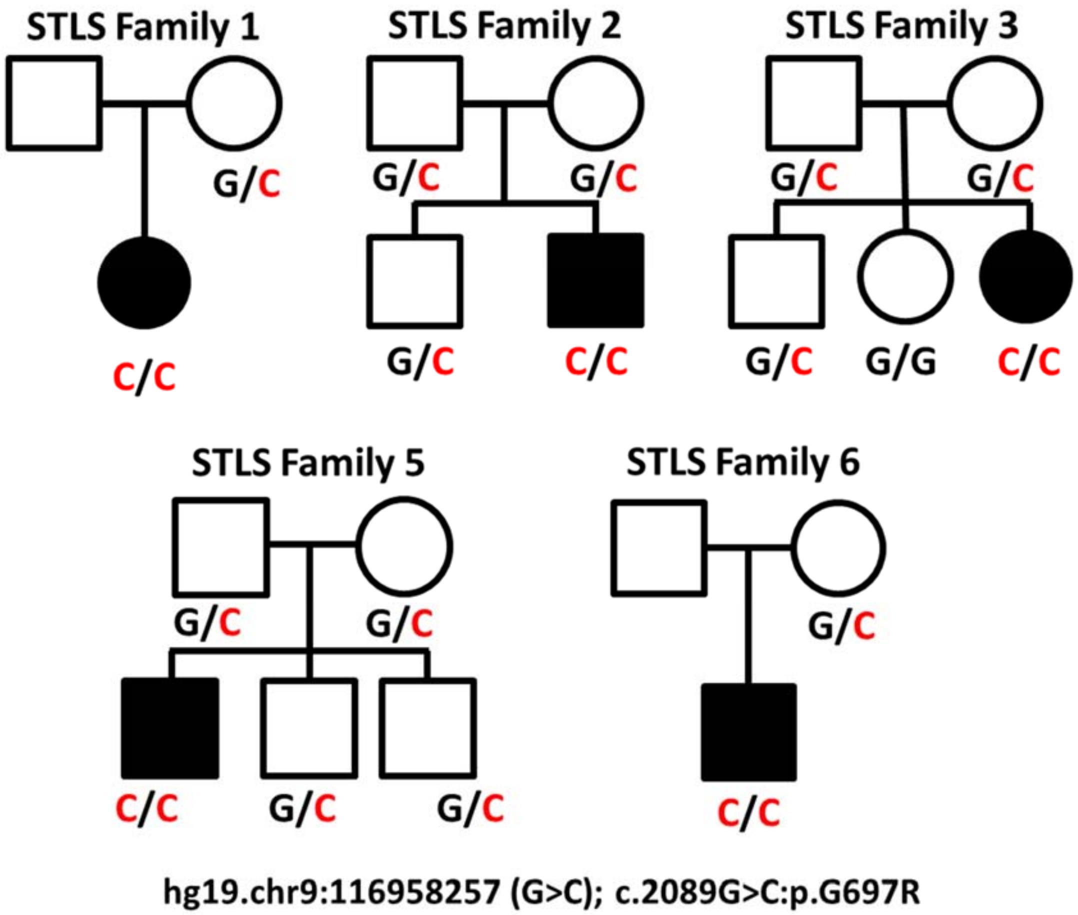


**Supplementary Figure 1.** Pedigrees of five of the original Steel Syndrome families reported by Steel et al (1993) and further characterized by Flynn et al (2010), where we confirmed appropriate segregation with affectation status for Steel syndrome and the G>C mutation at position chr9:116958257 (hg19) that produces the amino acid change p.Gly697Arg in COL27A1. Affected individuals depicted by full black symbols are homozygous for the variant (C/C), while unaffected individuals depicted as white symbols are heterozygous carriers (G/C) or homozygous reference (G/G).

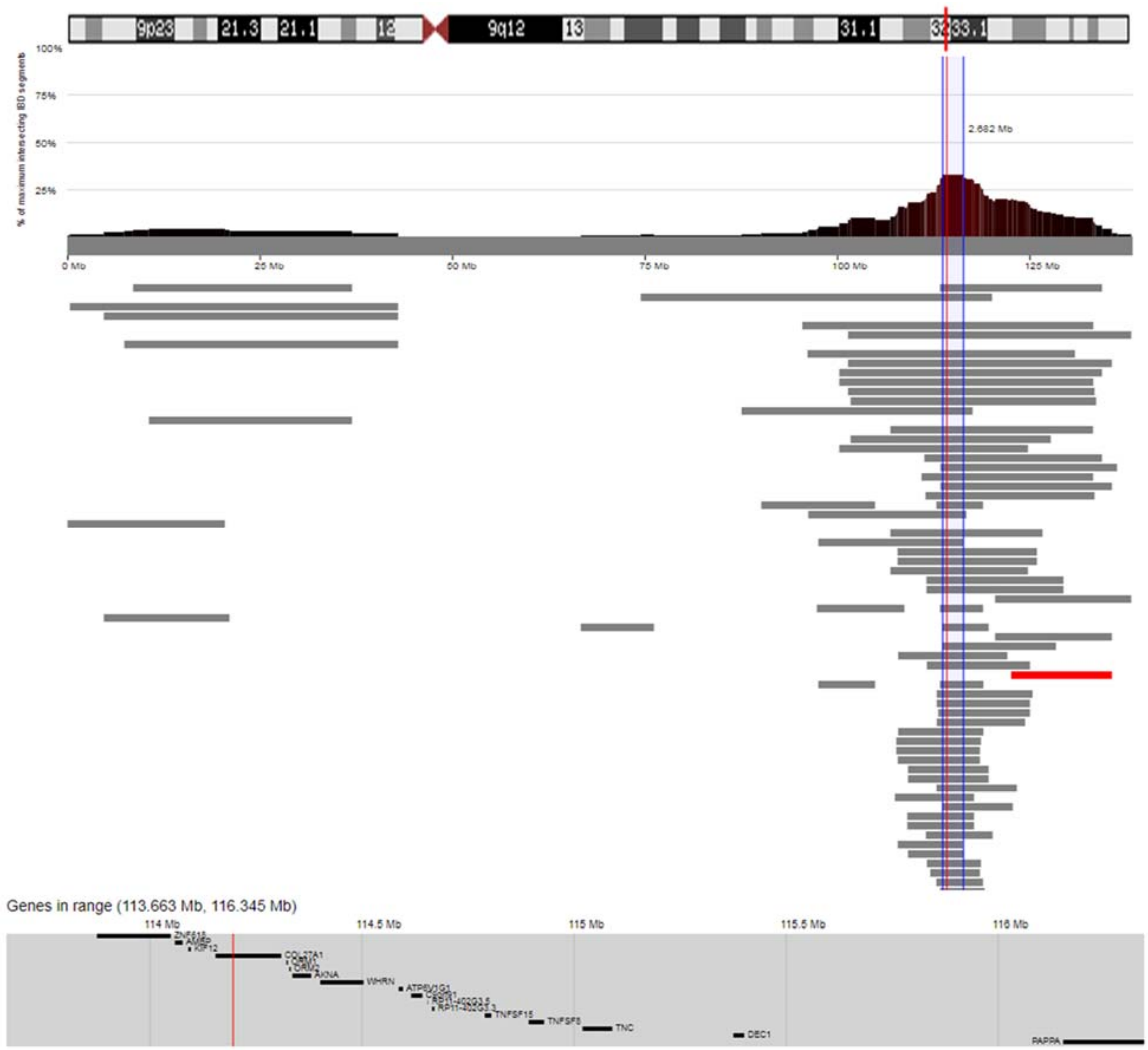


**Supplementary Figure 2. Identity-by-descent (IBD) segment mapping across heterozygous carriers of the c.2089G>C (p.Gly697Arg) variant in *COL27A1* in the DiscovEHR cohort.** Pairwise comparisons of shared IBD segments in heterozygous carriers of the STLS variant shows that the common founder haplotype in which the c.2089G>C (p.Gly697Arg) variant resides is 2.682Mb long and spans between 113.663Mb and 116.345Mb in chromosome 9.

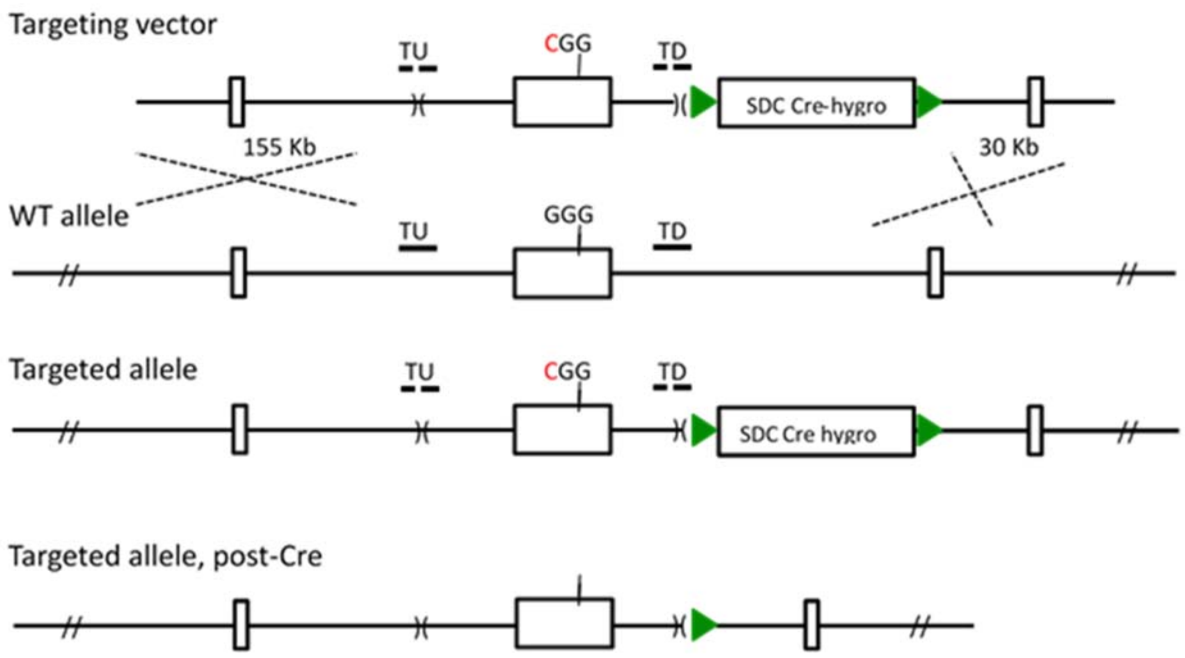


**Supplementary Figure 3. Targeting vector design and allele modification to generate *Col27a1* *(p.Gly682Arg)* knock-in mutant mice.** A DNA construct containing *Col27a1* exon 7 and surrounding intronic sequences was synthesized to include the desired G>C mutation, as well as two small intronic deletions to accommodate loss-of-allele (LOA) TaqMan assays TU and TD. An intermediate donor vector was created by cloning a floxed hygromycin resistance self-deleting cassette (SDC) into restriction sites introduced to intron 7. A murine BAC containing *Col27a1* was separately cleaved with two additional guides designed to create complementary overhangs with the donor vector. The donor and the BAC were combined together using Gibson assembly to create the final targeting vector. The final targeting vector was electroporated into C57BL/6N mouse embryonic stem cells and selected via hygromycin resistance in the self-deleting cassette.

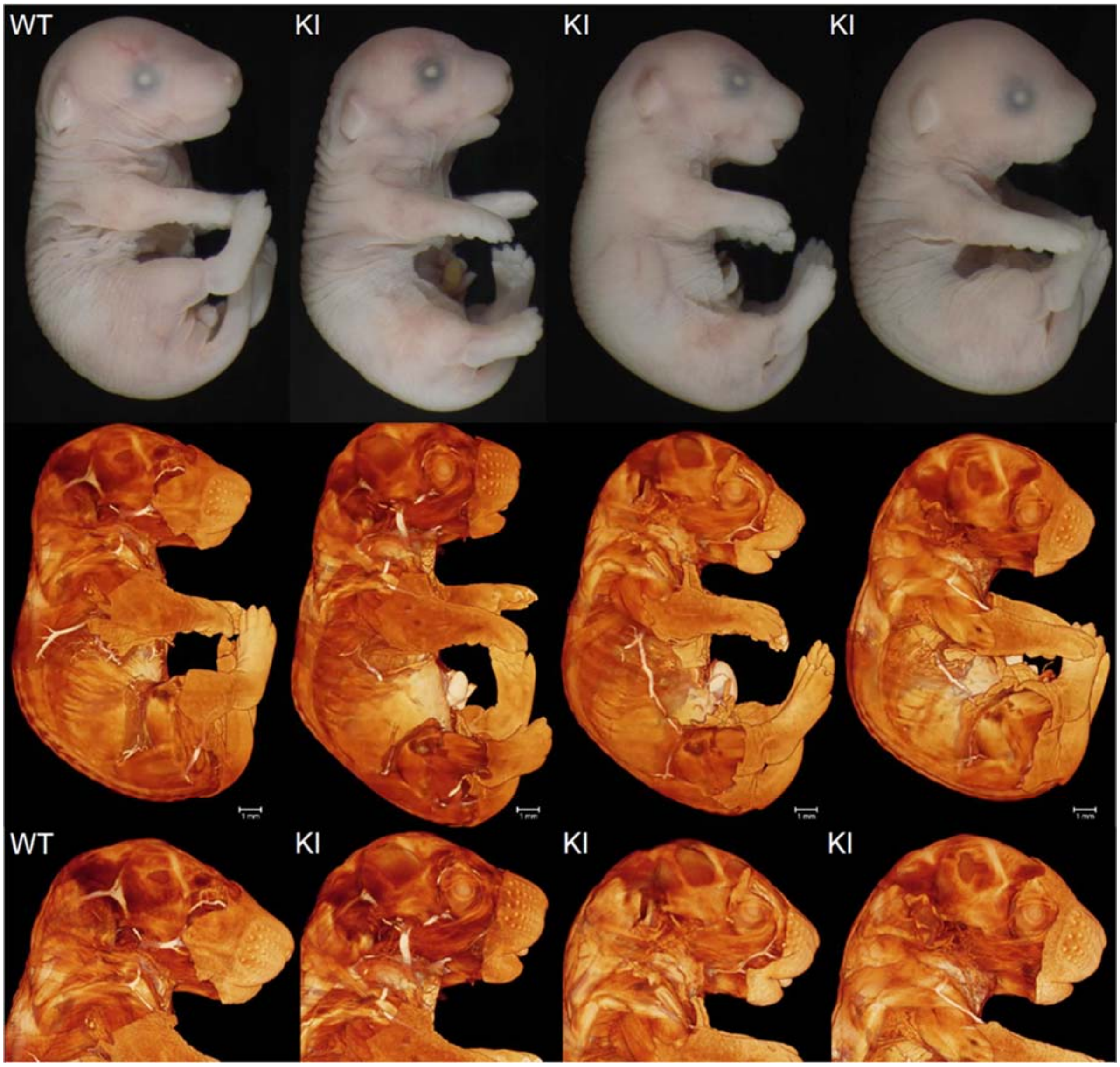


**Supplementary Figure 4. No gross abnormalities or size differences observed in *Col27a1* (G682R) KI versus WT mice during embryonic development.** Phenotyping of *Col27a1* homozygous KI embryos at E18.5 with microcomputed tomography imaging. No major abnormalities are noted at this stage, embryos are comparable in size to wild-type; however the skull in the homozygous mutant mice is rounder with a shorter snout as compared to wild-type and heterozygous KI embryos.

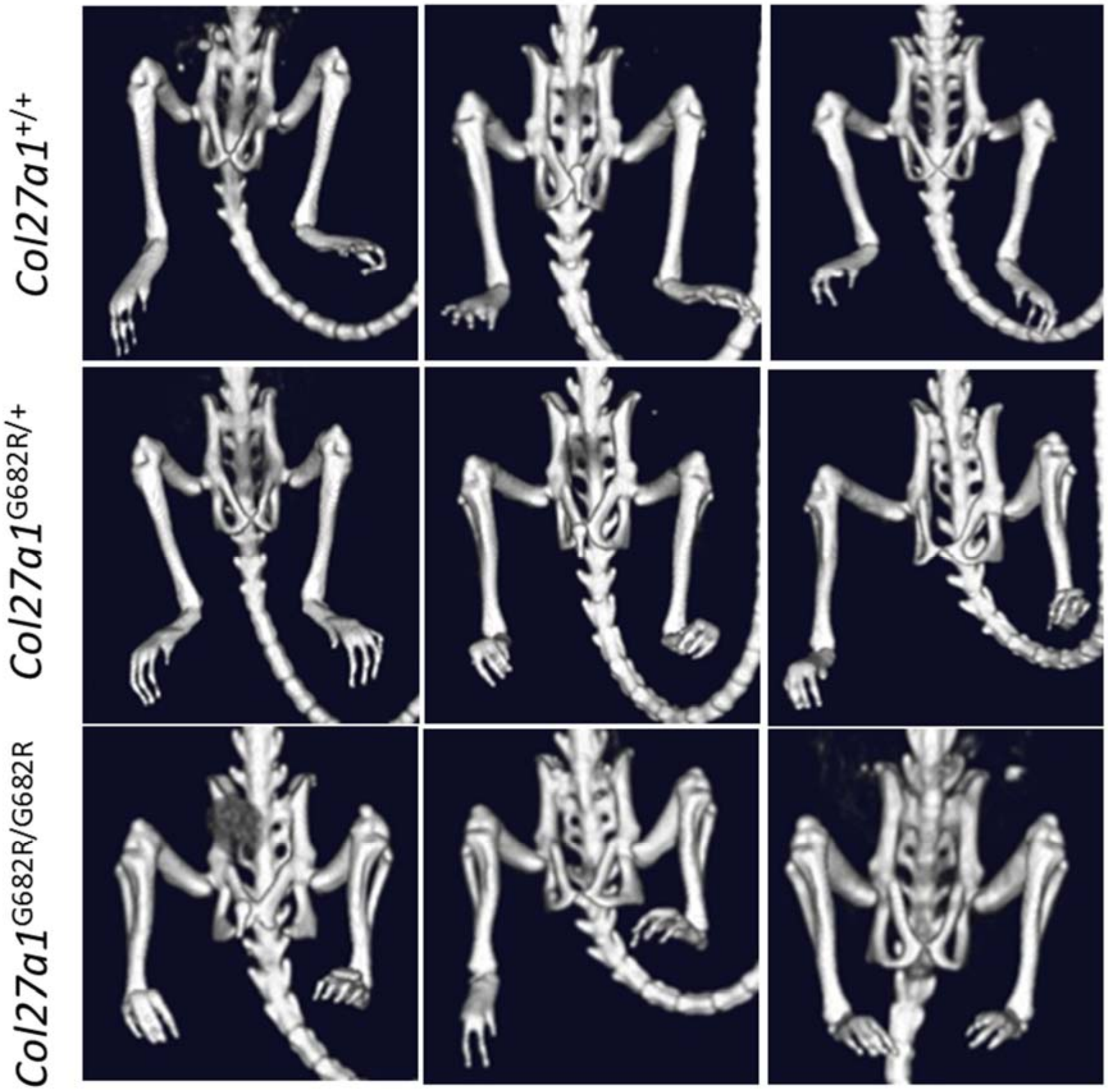


**Supplementary Figure 5. Varus deformity observed in *Col27a1* (G682R) KI mice.** Gross skeletal phenotyping of heterozygous *Col27a1*$^{G682R/+}$, homozygous *Col27a1*$^{G682R/G682R}$, and WT mice using µCT revealed that all the homozygous *Col27a1*$^{G682R/G682R}$ KI mice evaluated had varus deformity with in-toeing in both their hind limbs (n=6/7) or at least in one hind limb (n=1/7) (bottom panel); two thirds of heterozygous *Col27a1*$^{G682R/+}$ KI mice also displayed milder varus deformity of their hind limbs (middle panel), whereas none of WT littermates evaluated had varus deformity (top panel).

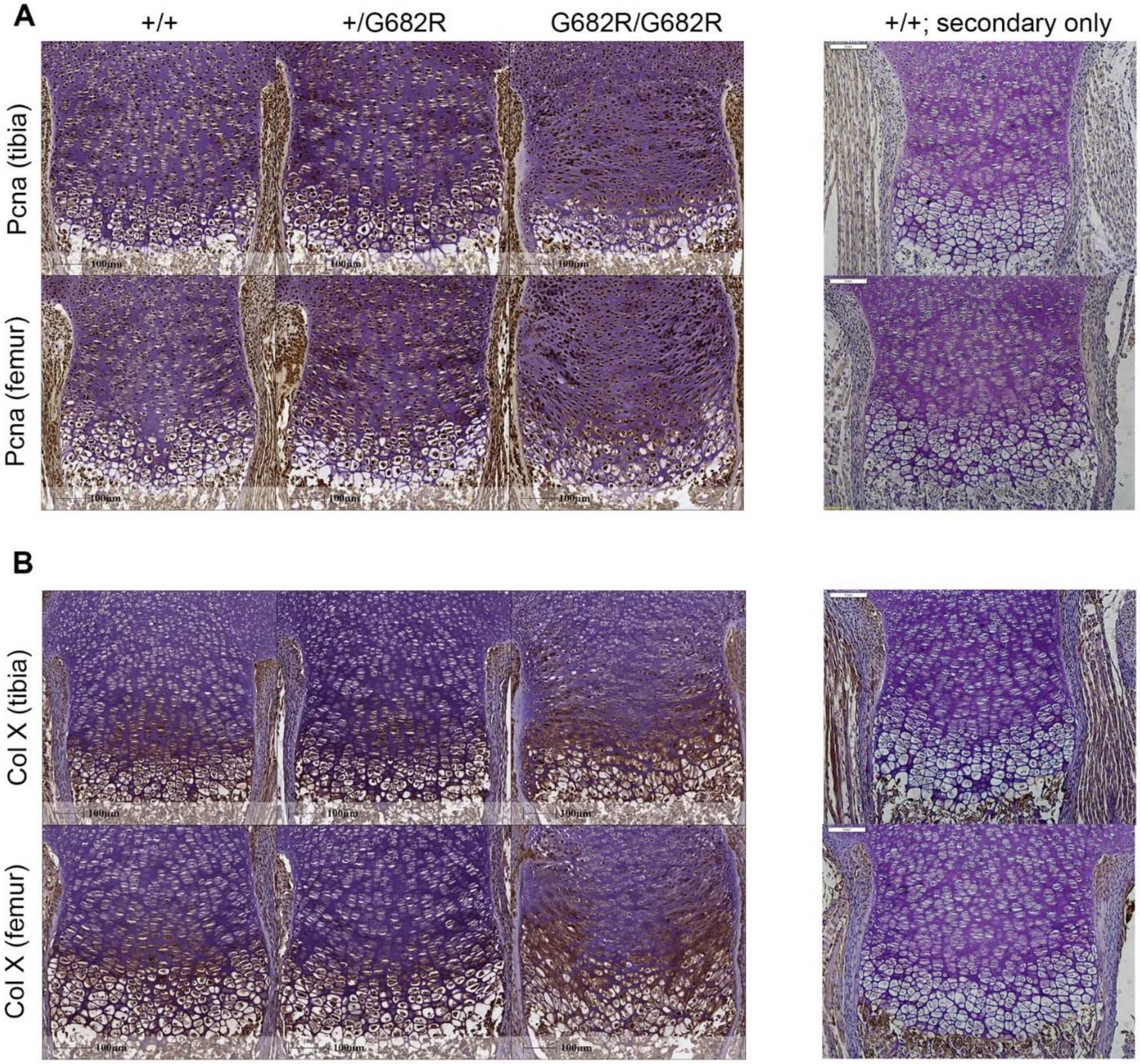


**Supplementary Figure 6. Immunohistochemical analysis of collagen distribution in *Col27a1* (G682R) KI mice.** (A & B) Longitudinal sections from post-natal day 1 growth plate of the developing tibia and femur of wild-type, heterozygous *Col27a1*$^{G682R/+}$, and homozygous *Col27a1*$^{G682R/G682R}$ KI mice. Sections are immunostained with (A) PCNA and (B) Collagen X. There is no overt difference in the staining intensity between genotypes. Disorganization in the columnar arrangement of hypertrophic chondrocytes can be observed in heterozygous *Col27a1*$^{G682R/+}$ and homozygous *Col27a1*$^{G682R/G682R}$ KI mice.

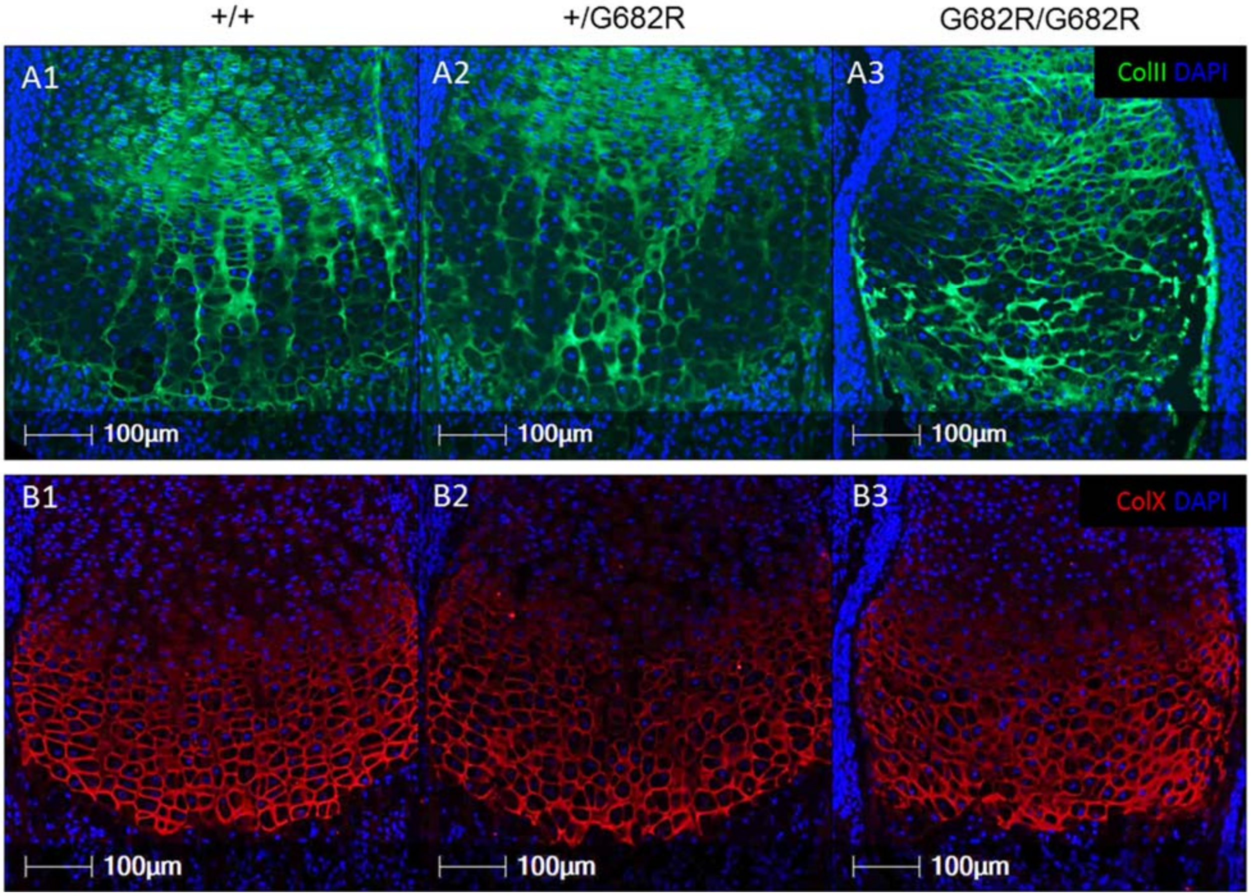


**Supplementary Figure 7. Immunohistochemical analysis of collagen distribution in *Col27a1* (G682R) KI mice.** (A & B) Longitudinal sections from post-natal day 1 growth plate of the developing tibia of wild-type, heterozygous *Col27a1*$^{G682R/+}$, and homozygous *Col27a1*$^{G682R/G682R}$ KI mice. Sections immunostained with (A) Collagen II and (B) Collagen X and counterstained with DAPI for cell nuclei. There is no overt difference in the staining intensity between genotypes. Disorganization in the columnar arrangement of hypertrophic chondrocytes can be observed in heterozygous *Col27a1*$^{G682R/+}$ and homozygous *Col27a1*$^{G682R/G682R}$ KI mice.

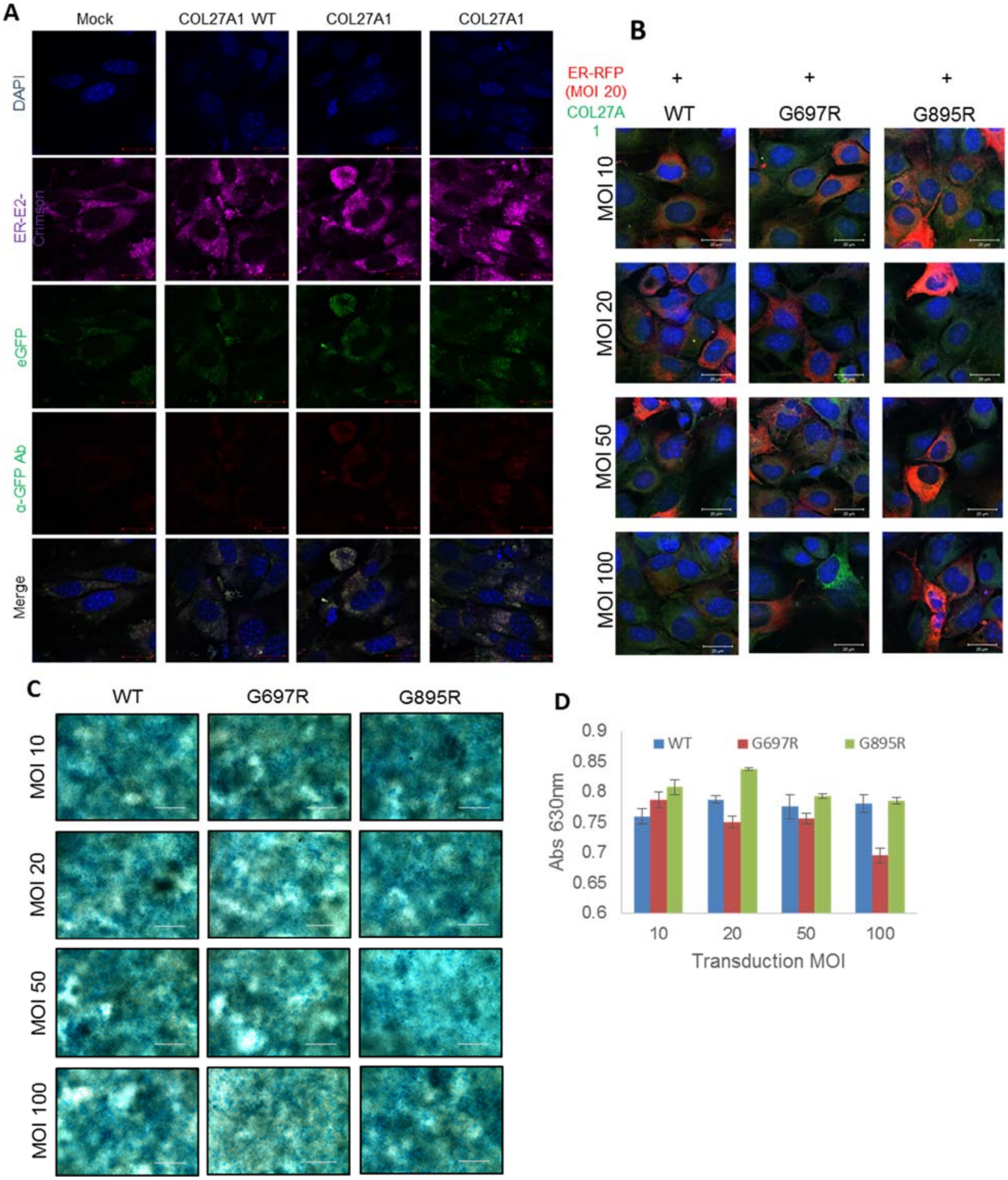
A
Mock
COL27A1 WT
COL27A1
COL27A1
DAPI
ER-E2-Crimson
eGFP
α-GFP Ab
Merge
B
ER-RFP (MOI 20)
COL27A1
+
+
+
WT
G697R
G895R
MOI 10
MOI 20
MOI 50
MOI 100
C
WT
G697R
G895R
MOI 10
MOI 20
MOI 50
MOI 100
D
WT
G697R
G895R
Abs 630nm
0.9
0.85
0.8
0.75
0.7
0.65
0.6
10
20
50
100
Transduction MOI

**Supplementary Figure 8. The Gly697Arg and Gly895Arg variants possibly result in ER retention of mutant COL27A1.** (A) Representative confocal images of W20 cells overexpressing c-terminal GFP-tagged COL27A1-WT, G697R and G895R constructs, ER-crimson is co-expressed to visualize ER (scale bar: 20µM). (B) Representative confocal images of adenoviral transduced ATDC5 cells overexpressing c-terminal GFP-tagged COL27A1-WT, G697R and G895R at different multiplicities of infection (10-100) per cell, ER-RFP is co-expressed (at MOI 20) to visualize ER (scale bar: 20µM). (C) Chondrogenic differentiation of ATDC5 cells overexpressing WT, G697R and G895R variants represented by proteoglycan accumulation using alcian blue staining (scale bar: 1000µM), spectrophotometric quantification of alcian blue staining is represented in the histogram below (D).

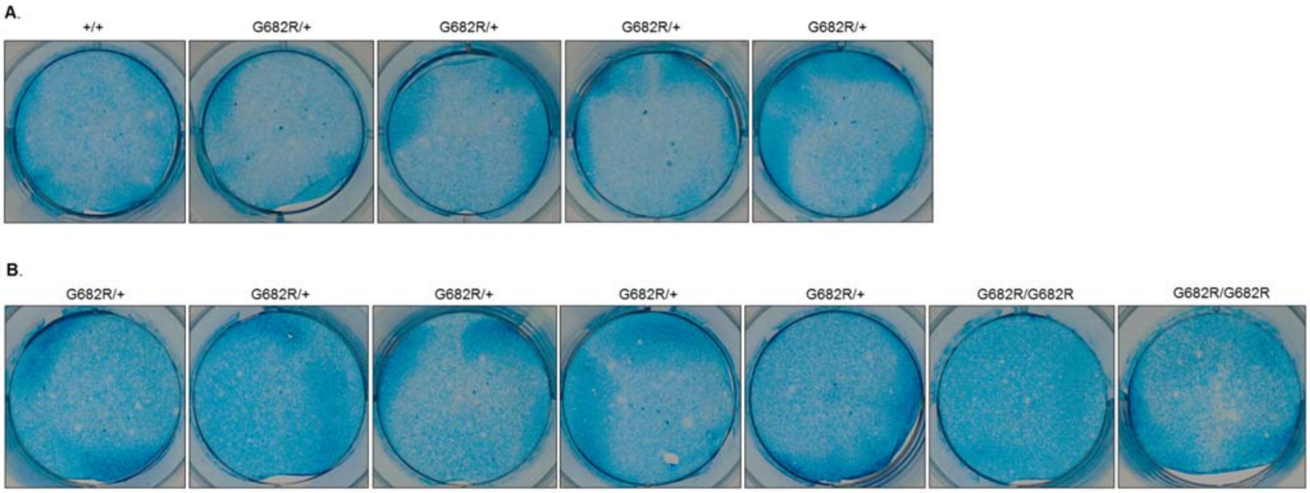


**Supplementary Figure 9. Orthologous p.Gly682Arg mutation has no cell-autonomous effects on chondrogenic differentiation *in vitro*.** Primary chondrocytes derived from heterozygous *Col27a1*$^{G682R/+}$ or homozygous *Col27a1*$^{G682R/G682R}$ KI mice show no differences in chondrocyte differentiation as compared to wild-type littermates based on proteoglycan accumulation using alcian blue staining. Chondrogenic differentiation of primary chondrocytes derived from two independent litters (A and B).

**Supplementary Table 1.** Demographics and clinical diagnoses of interest for 29 adult carriers of the STLS (c.2089G>C; p.Gly697Arg) variant in COL27A1 in the DiscovEHR cohort, including an individual homozygous for the STLS variant (Patient GHS01, highlighted in blue) that presents with the classical clinical characteristics of Steel syndrome.

| Patient | Age (y) | Sex | Reported Race | Reported Ethnicity | Height (m) | Weight (kg) | BMI | Diagnoses_of_Interest | G697R |
|---|---|---|---|---|---|---|---|---|---|
| **GHS01** | 39 | F | White | Hispanic or Latino | 1.50 | 51.7 | 23.0 | Abnormal MRI, cervical spine; Broken internal left hip prosthesis; Cervical radiculopathy; Elbow deformity; Failure of total hip arthroplasty; Fracture of other bone following insertion of orthopedic implant, joint prosthesis, or bone plate; Gait abnormality; Hip pain; History of congenital dysplasia of hip; Left foot drop; Leg length discrepancy; Mechanical loosening of internal left hip prosthetic joint, sequela; Numbness and tingling of left leg; Other idiopathic scoliosis, unspecified spinal region; Pain of both elbows; Pain of both shoulder joints; Pain of left lower leg; Pain of lower extremity; Presence of artificial hip joint; Radial head subluxation, unspecified laterality; Sciatica of left side; Sensorineural hearing loss (SNHL) of both ears; Tinnitus of both ears; Wear of articular bearing surface of internal prosthetic left hip joint | Hom |
| **GHS02** | 51 | F | White | Hispanic or Latino | 1.45 | 54.0 | 25.8 | Acute right ankle pain; Backache; Cervicalgia; Pain in joint; Pain in limb; Pain in left knee; Shoulder joint pain | Het |
| **GHS03** | 63 | F | White | Hispanic or Latino | 1.52 | 82.5 | 35.5 | Degenerative disc disease; Pain in both feet; Pain of right upper arm | Het |
| **GHS04** | 63 | F | White | Hispanic or Latino | 1.55 | 66.7 | 27.8 | . | Het |
| **GHS05** | 32 | F | White | Hispanic or Latino | 1.55 | 73.5 | 30.6 | Ankle joint pain; Degenerative disc disease, lumbar; Hip pain; Low back pain; Radiculitis; Temporomandibular joint disorders | Het |
| **GHS06** | 29 | F | White | Hispanic or Latino | 1.55 | 68.0 | 28.3 | Knee pain; Pain in joint; Pain in left hand and shoulder | Het |
| **GHS07** | 52 | F | White | Hispanic or Latino | 1.57 | 88.0 | 35.5 | Arthralgia; Arthropathy; Bilateral shoulder bursitis; Cervicalgia; Carpal tunnel syndrome; Costochondritis; Degeneration of lumbosacral intervertebral disc; Elbow pain; Hand joint stiff; Joint pain; Other chronic pain; Pain in joint; Pain in limb; Rheumatoid arthritis of multiple sites | Het |
| **GHS08** | 42 | F | White | Hispanic or Latino | 1.57 | 126.5 | 51.0 | . | Het |
| **GHS09** | 31 | F | Native Hawaiian Or Other Pacific Islander | Hispanic or Latino | 1.57 | 69.2 | 27.9 | Wrist tendonitis | Het |
| **GHS10** | 29 | F | White | Hispanic or Latino | 1.60 | 98.4 | 38.4 | Pain in limb | Het |

| Patient | Age (y) | Sex | Reported Race | Reported Ethnicity | Height (m) | Weight (kg) | BMI | Diagnoses_of_Interest | G697R |
|---|---|---|---|---|---|---|---|---|---|
| **GHS11** | 47 | F | White | Not Hispanic or Latino | 1.63 | 57.6 | 21.8 | Backache; Chronic back pain | Het |
| **GHS12** | 27 | F | White | Not Hispanic or Latino | 1.63 | 84.4 | 31.9 | Joint pain, hip; Pelvic pain in female; Sprain of hip and thigh | Het |
| **GHS13** | 27 | M | White | Not Hispanic or Latino | 1.63 | 73.9 | 28.0 | Bilateral club feet; Curvature of spine associated with other condition; Idiopathic scoliosis; In-toeing; Pain in limb | Het |
| **GHS14** | 61 | M | White | Hispanic or Latino | 1.65 | 62.1 | 22.8 | Backache; Hand joint pain; Localized, primary osteoarthritis of hand; Osteoarthrosis; Pain in limb; Primary localized osteoarthrosis, lower leg | Het |
| **GHS15** | 54 | M | White | Hispanic or Latino | 1.65 | 113.4 | 41.6 | Arthralgia of hands, bilateral; Back pain; Cervicalgia; Left elbow pain; Neck pain; Pain of left upper extremity; Right elbow pain | Het |
| **GHS16** | 52 | M | White | Hispanic or Latino | 1.65 | 110.7 | 40.6 | Arthralgia of both hands; Back pain; Knee pain; Patellofemoral syndrome; Pain in joint of right shoulder | Het |
| **GHS17** | 37 | F | White | Hispanic or Latino | 1.65 | 87.1 | 32.0 | Low back pain | Het |
| **GHS18** | 28 | F | White | Not Hispanic or Latino | 1.65 | 112.0 | 41.1 | No data available | Het |
| **GHS19** | 59 | F | White | Hispanic or Latino | 1.68 | 91.6 | 32.6 | Acute pain of right knee; Arthralgia; Chronic pain of both knees; Osteoarthritis of both knees; Pain in joint, multiple sites; Pain in limbs; Polyarthralgia; Polyarthritis; Primary osteoarthritis of both knees; Radiculopathy, lumbar region | Het |
| **GHS20** | 23 | F | White | Hispanic or Latino | 1.68 | 93.4 | 33.3 | Left knee pain; patellar subluxation | Het |
| **GHS21** | 50 | F | White | Hispanic or Latino | 1.70 | 78.5 | 27.1 | Acute pain of left and right knee; Left foot pain; Other idiopathic scoliosis, thoracic region; Pain in joint of right shoulder | Het |
| **GHS22** | 21 | F | White | Not Hispanic or Latino | 1.70 | 84.8 | 29.3 | Back pain; Hammer toe; Joint pain, knee; | Het |
| **GHS23** | 42 | M | White | Hispanic or Latino | 1.73 | 99.3 | 33.3 | Arthralgia; Degenerative joint disease; Pain in joint; Right shoulder pain | Het |
| **GHS24** | 76 | M | White | Hispanic or Latino | 1.75 | 81.9 | 26.7 | Pain in both feet; Pain in limb | Het |
| **GHS25** | 47 | M | White | Hispanic or Latino | 1.78 | 83.9 | 26.5 | Backache; Cervicalgia; Low back pain; Osteoarthrosis | Het |
| **GHS26** | 46 | M | White | Hispanic or Latino | 1.78 | 76.2 | 24.1 | Backache; Disorder of intervertebral disc; Multiple joint pain; | Het |
| **GHS27** | 59 | M | White | Hispanic or Latino | 1.79 | 133.3 | 41.8 | Degeneration of lumbar or lumbosacral intervertebral disc; Osteoarthrosis; Osteoarthritis; Rheumatoid arthritis | Het |
| **GHS28** | 39 | M | White | Hispanic or Latino | 1.83 | 101.6 | 30.4 | Arthropathy; Ankle pain, chronic, right; Pain in joint, knee | Het |
| **GHS29** | 34 | M | White | Not Hispanic or Latino | 1.83 | 99.8 | 29.8 | Hand joint pain; Knee pain; Pain in limb; Rotator cuff syndrome; Shoulder joint pain | Het |

*F: Female; M: Male. Hom: Homozygous for the c.2089G>C (p.Gly697Arg) variant in COL27A1; Het: Heterozygous for the c.2089G>C (p.Gly697Arg) variant in COL27A1.*
